# A computational model of cardiomyocyte metabolism predicts unique reperfusion protocols capable of reducing cell damage during ischemia/reperfusion

**DOI:** 10.1016/j.jbc.2022.101693

**Published:** 2022-02-11

**Authors:** Matthias Grass, Anthony D. McDougal, Adriana Blazeski, Roger D. Kamm, Guillermo García-Cardeña, C. Forbes Dewey

**Affiliations:** 1 Department of Mechanical Engineering, ETH Zurich, Zurich, Switzerland; 2 Department of Pathology, Brigham and Women’s Hospital, Boston, Massachusetts, USA; 3 Program in Human Biology and Translational Medicine, Harvard Medical School, Boston, Massachusetts, USA; 4 Department of Mechanical Engineering, Massachusetts Institute of Technology, Cambridge, Massachusetts, USA; 5 Department of Biological Engineering, Massachusetts Institute of Technology, Cambridge, Massachusetts, USA; 6 Cardiovascular Disease Initiative, The Broad Institute of MIT and Harvard, Cambridge, Massachusetts, USA

**Keywords:** ischemia/reperfusion injury, computational modeling, cardiomyocyte metabolism, biochemical modeling, mitochondrial membrane complexes, reactive oxygen species, oxidative damage, ODE, ordinary differential equation, RET, reverse electron transport, ROS, reactive oxygen species, TCA, tricarboxylic acid

## Abstract

If a coronary blood vessel is occluded and the neighboring cardiomyocytes deprived of oxygen, subsequent reperfusion of the ischemic tissue can lead to oxidative damage due to excessive generation of reactive oxygen species. Cardiomyocytes and their mitochondria are the main energy producers and consumers of the heart, and their metabolic changes during ischemia seem to be a key driver of reperfusion injury. Here, we hypothesized that tracking changes in cardiomyocyte metabolism, such as oxygen and ATP concentrations, would help in identifying points of metabolic failure during ischemia and reperfusion. To track some of these changes continuously from the onset of ischemia through reperfusion, we developed a system of differential equations representing the chemical reactions involved in the production and consumption of 67 molecular species. This model was validated and used to identify conditions present during periods of critical transition in ischemia and reperfusion that could lead to oxidative damage. These simulations identified a range of oxygen concentrations that lead to reverse mitochondrial electron transport at complex I of the respiratory chain and a spike in mitochondrial membrane potential, which are key suspects in the generation of reactive oxygen species at the onset of reperfusion. Our model predicts that a short initial reperfusion treatment with reduced oxygen content (5% of physiological levels) could reduce the cellular damage from both of these mechanisms. This model should serve as an open-source platform to test ideas for treatment of the ischemia reperfusion process by following the temporal evolution of molecular concentrations in the cardiomyocyte.

A major cause of death globally, ischemic heart disease, is caused by a narrowing of the coronary arteries over time (1). Tissues become ischemic when blood vessels are not able to provide them with sufficient nutrients and oxygen (2). A myocardial infarction caused by a major cessation of coronary blood flow due to arterial dysfunction is a dramatic example of the onset of ischemia. The drop in intracellular oxygen levels in the cardiac muscle cells leads to impaired energy (ATP) production and an inability to sustain contractile function (3). Depending upon the degree and duration of oxygen loss, the affected heart muscle cells can die. The most obvious countermeasure to ischemia is the timely reperfusion of the affected vessel and restoration of normal oxygen levels. The availability of oxygen induces a reactivation of the electron transport chain and production of ATP within the mitochondria.

Paradoxically, conditions present during ischemia prime the tissue for significant damage upon reperfusion depending on the duration and severity of the ischemic period (4, 5, 6). Excessive amounts of reactive oxygen species (ROS) are produced at various sites in the mitochondria (7, 8). In the mitochondria's outer membrane, monoamine oxidase (MAO) and cytochrome b5 reductase (Cb5R) can produce ROS (9). In the inner mitochondrial membrane, glycerol-3-phosphate dehydrogenase, linked to the CoQ pool, can contribute to ROS production (8, 10). Other enzymes that interact with the mitochondrial NADH pool, such as αKDGH (α-ketoglutarate dehydrogenase) and other enzymes of the tricarboxylic acid (TCA) cycle (9), can contribute to superoxide production in the mitochondrial matrix (8, 11). Finally, mitochondrial enzymes not associated with the NADH or CoQ pools, such as cytochrome P450 enzymes, can also generate ROS in the mitochondria (12), as can NOX4, an NADPH oxidase that produces hydrogen peroxide, although its presence in the mitochondria is debated (13, 14, 15). Importantly, mitochondrial protein complexes I and III have been identified as the main ROS producers during reperfusion. Chouchani *et al.* (16) have shown how the accumulation of the Krebs cycle intermediary succinate during ischemia leads to a reversal of the electron transport chain at complex I and consequently to an excessive superoxide production and oxidative damage. On the other hand, Korge *et al.* (17) suggest that reverse electron transport (RET) is only partially responsible for the oxidative damage during reperfusion and highlight the superoxide production at complex III as a key driver of ROS production and cell damage. Other data have been put forward to justify the failure of calcium regulation to be a driving force for dysfunction of the mitochondria (18).

Although many studies have identified the important role of the mitochondria in ischemia/reperfusion injury (19, 20, 21), few have quantitatively shown the mechanisms and conditions present during the initial moments of reperfusion. These initial conditions require a careful assessment of the cumulative effects of ischemia and the time-dependent evolution of molecular components of the cardiomyocyte and its mitochondria. Analytical models should include the time course of restarting blood flow as this may be crucial to the fate of the cardiomyocytes. McDougal and Dewey (22) created a mathematical model of the anaerobic cardiomyocyte metabolism during the transition from a normal physiological state to one dominated by ischemia. This study extends that model using a biochemical description of the mitochondria that includes some aspects of oxidative phosphorylation and compartmental transport processes, inspired by the work of Beard (23). The resulting model predicts the metabolic changes during the critical transition periods between ischemia and reperfusion and, additionally, suggests novel reperfusion protocols to minimize injury from oxidative damage.

## Results

### Static experiments (physiological steady state and sensitivity)

**Figure 1:**
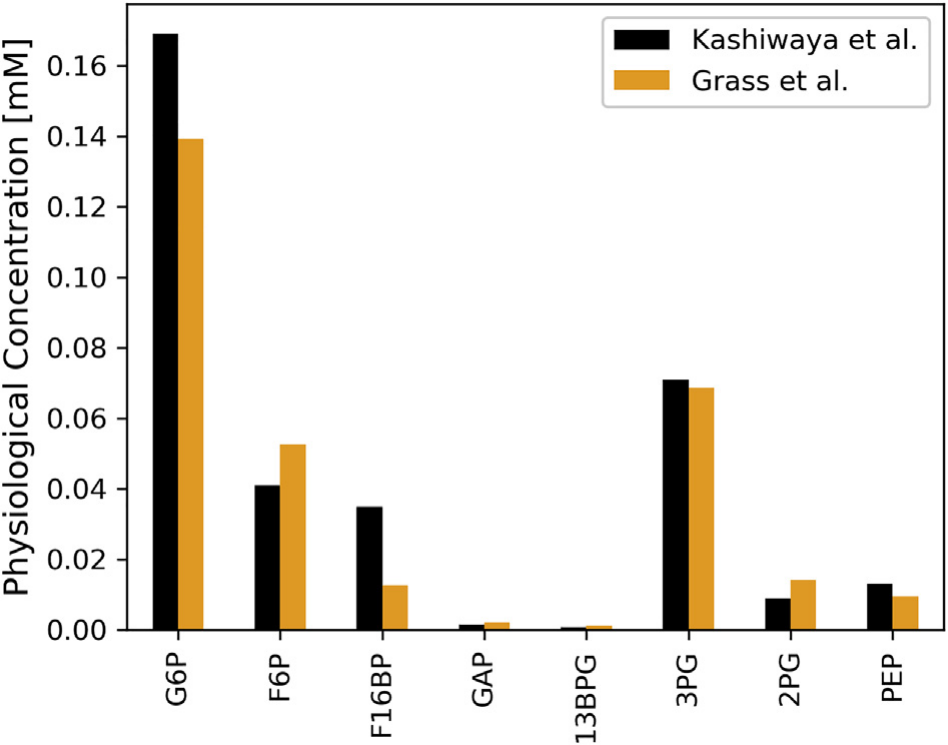
**Validation of glycolytic intermediates.** Comparison of normal physiology steady-state concentrations between model predictions (*orange*) and values obtained experimentally in perfused rat hearts (*black*) (24).

In order to validate the predictions of our metabolic model, the chemical concentrations of intracellular species related to the glycolytic pathway were computed until a chemical steady state was reached. These values were then compared with experimental results from Kashiwaya *et al.* (24), who measured glycolytic intermediates in perfused rat hearts (see Fig. 1).

Simultaneously, a sensitivity analysis was conducted, where the dependence of chemical concentrations with respect to enzyme activities was computed and quantitatively compared with experimental observations. More specifically, the relative sensitivity of species i with respect to the activity of enzyme j was calculated as the relative change in concentration of species i due to a 1% change in activity of enzyme j. These values were then summed over all species to quantify the influence of a given enzyme/reaction. The summed sensitivity values associated with enzyme *j* are represented by *S*_*j*_. Table 1 shows the qualitative distribution of control across the metabolic network and highlights the regulation of ATP levels through multiple mechanisms. The aggregated sensitivity values highlight the importance of the reaction catalyzed by hexokinase for the downstream part of the glycolytic pathway during physiological conditions. With reduced activity of hexokinase, the amount of G6P will be reduced and the cardiac glucose metabolism is expected to slow down. However, Table 1 also shows the distribution of metabolic control among multiple pathways other than glycolysis. While, during ischemia, the sensitivity values are led by the activity of glucose-6-phosphate dehydrogenase, after reperfusion the sensitivity values are very similar to the preischemic values due to the absence of any irreversible processes implemented in our model. The comparison of the sensitivity values reported in Table 1 with experimental studies regarding the distribution of metabolic control in cardiomyocytes such as reported by Kashiwaya *et al.* (24) further serves as a qualitative validation of our model. For a mathematical explanation of these sensitivity coefficients, please refer to section Simulations/Sensitivity analysis in Computational Methods.

**Table 1:** Enzymes/reactions exerting the largest overall control

Perfusion state	Enzyme/reaction	Pathway	S	Rank
Physiol	HK	Glycolysis	16.54	1
	ANT	ATP/ADP Transporter	10.73	2
	ATPase	ATP Consumption	10.19	3
	PFK	Glycolysis	6.93	4
	G6PDH	Pentose Phosphate	4.43	5
Ischemia	G6PDH	Pentose Phosphate	17.16	1
	HK	Glycolysis	15.58	2
	PFK	Glycolysis	5.83	3
	TPI	Glycolysis	4.57	4
	AK	Energy Buffer	4.31	5
Reperfusion	HK	Glycolysis	16.36	1
	ANT	ATP/ADP Transporter	10.80	2
	ATPase	ATP Consumption	8.29	3
	PFK	Glycolysis	7.36	4
	G6PDH	Pentose Phosphate	4.29	5

Values are L1-norm of the relative sensitivities across metabolites.

### Dynamic validations (ischemia and reperfusion transitions)

**Figure 2:**
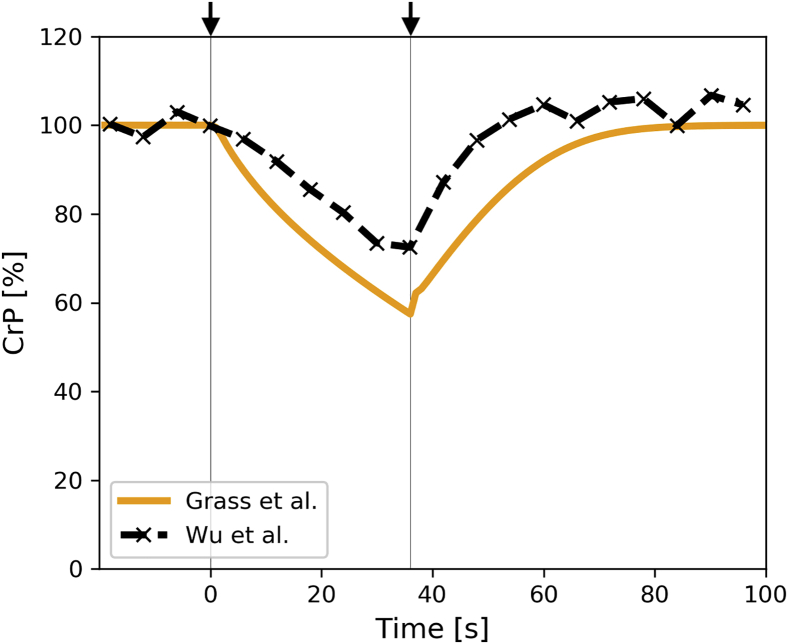
**Validation of ischemia and reperfusion transitions in canine hearts.** Comparison of cytosolic creatine phosphate concentrations between model predictions (*orange*) and values obtained experimentally in canine hearts subject to 36 s of ischemia and 64 s of reperfusion. For ischemia simulations, extracellular oxygen concentration was set to 1% of the physiological level.

To compare model predictions with published experimental results, simulations were carried out using different values for ischemic oxygen concentrations and time durations of the ischemic period. First, results were compared with ischemic canine hearts (25), where creatine phosphate was measured during 36 s of ischemia (left arrow indicates start of ischemia, right arrow indicates end of ischemia) for 100 s in total (see Fig. 2).

**Figure 3:**
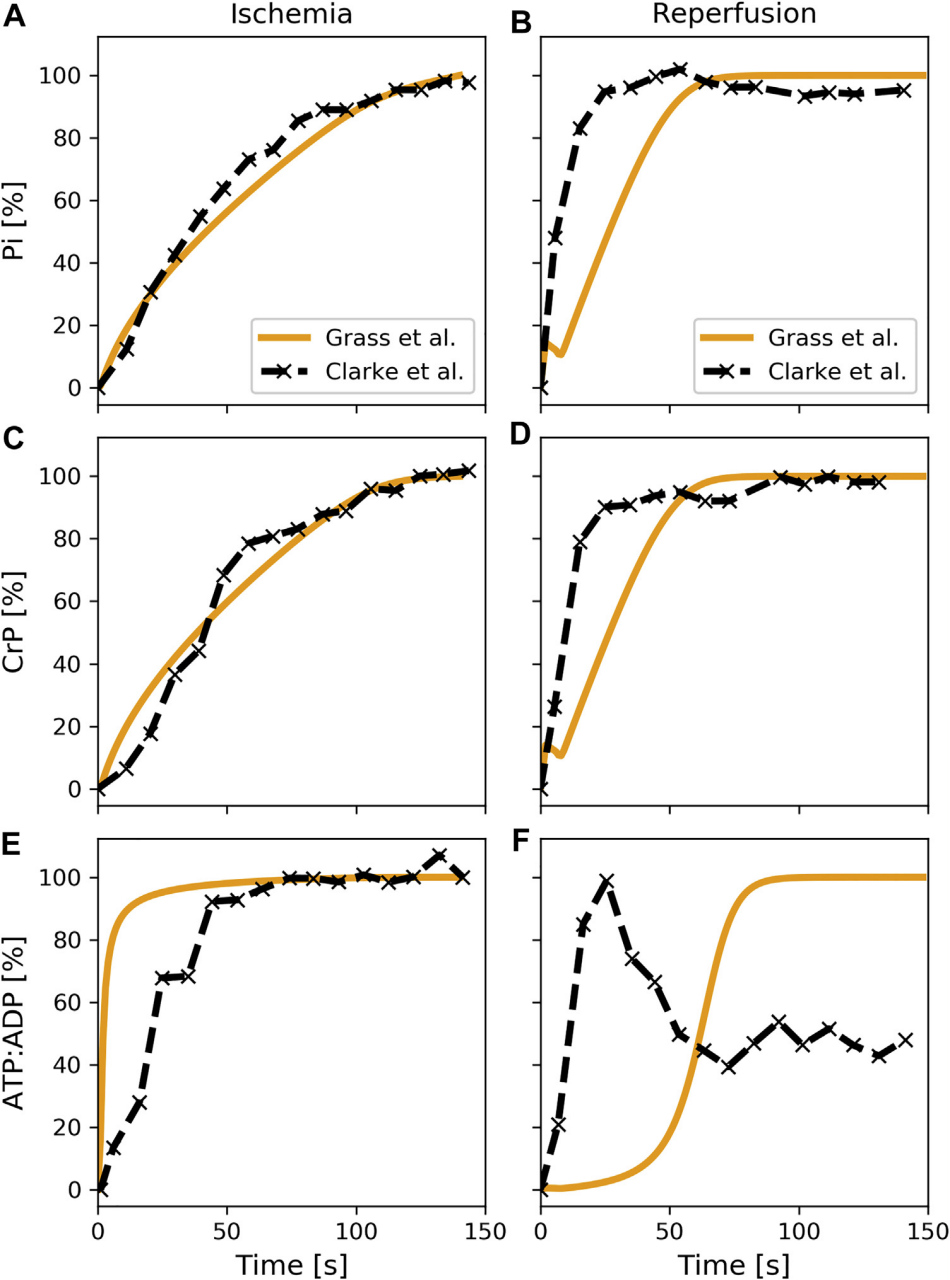
**Validation of ischemia and reperfusion transitions in guinea pig hearts.** Comparison of cytosolic phosphate metabolite concentrations between model predictions (*orange*) and values obtained experimentally in guinea pig hearts subject to 150 s of ischemia (1% of physiological oxygen) and 150 s of reperfusion. The y-axes are normalized to represent % of the total change over 150 s, as reported by Clarke *et al.* (26) (*e.g.*, $Pi_{rel}\left(t\right)=\frac{\left|Pi\left(t\right)-Pi_{min}\right|}{\left|Pi_{max}-Pi_{min}\right|}\cdot 100$). ATP:ADP indicates the ratio of ATP to ADP, a common readout to determine the metabolic state of the cardiomyocyte (87). *A*, relative change in cytosolic inorganic phosphate (Pi) in simulations correlates with experimental measurements during ischemia. *B*, reperfusion simulations of inorganic phosphate (Pi) also show correlation with experiments. Experimental measurements reach peak slightly faster than simulations. *C*, relative change in cytosolic creatine phosphate (CrP) in simulations correlates with experimental measurements during ischemia. *D*, similar to phosphate measurements, simulated changes in creatine phosphate (CrP) during reperfusion show correlation with experiments but xperimental measurements reach peak concentrations faster (~50 s) than simulations (~60 s). *E*, relative change in ratio of cytosolic ATP:ADP in simulations correlates again with experimental measurements. Ratio saturates faster in simulations than in experimental measurements. *F*, simulated ATP:ADP ratio during reperfusion shows different behavior than experiments. While simulation shows an S-curve with an inflection point at around ~60 s, experiments show a fast saturation of ATP:ADP ratio before stabilizing at ~50%.

Additionally, results were compared with experimental measurements by Clarke *et al.* (26), where ischemia was introduced in guinea pig hearts for 150 s, and phosphate-related species were measured for the 150 s of ischemia and 150 s of reperfusion (see Fig. 3).

### Oxygen dependence of ischemia/reperfusion

**Figure 4:**
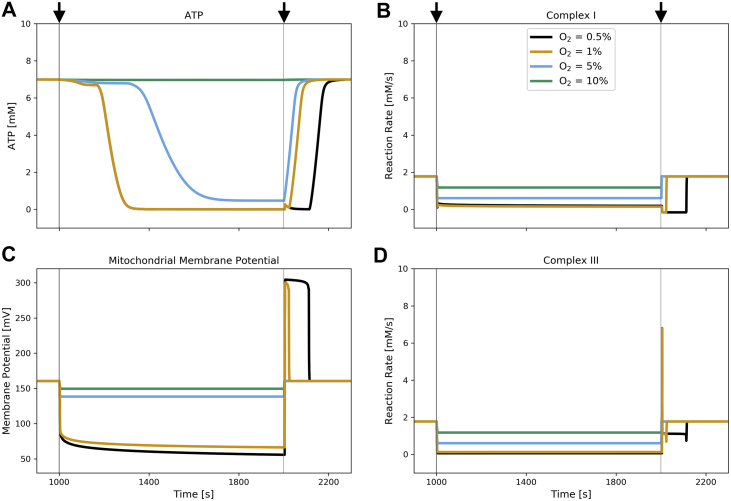
**Ischemia/reperfusion simulations for different levels of residual ischemic oxygen concentrations.** Oxygen dependence of metabolic measurements during ischemia (*first vertical lines*; *left arrows*) and reperfusion (*second vertical lines*; *right arrows*). Oxygen levels in legend refer to values in percent of normal physiological level. *A*, molecular concentration of ATP, (*B*) reaction rate at complex I of the electron transport chain (*i.e.*, the amounts of NADH and Q are converted to NAD and QH2 per second; see Equation 33), (*C*) mitochondrial membrane potential, (*D*) reaction rate at complex III (*i.e.*, the amounts of QH2 and cytochrome C (oxidized) are converted to Q and cytochrome C (reduced) per second; see Equation 35).

Even if a blood vessel is fully occluded, the neighboring tissue will always have a small residual perfusion of other nonoccluded blood vessels. Thus, the extracellular oxygen concentration will likely not reach absolute 0. Model simulations were carried out at decreased oxygen concentrations between 10% and 0.5% of normal physiological levels, uncovering trends for the cellular mechanisms that correlate with the severity of ischemia (see Fig. 4). The total simulation time was 3000 s, while introducing varying degrees of ischemia at t = 1000 s and reintroducing preischemic oxygen levels at t = 2000 s. Surprisingly, simulation results indicate that the ATP production in cardiomyocytes can meet demand at oxygen levels as low as 10% of preischemic levels for a sustained duration (see Fig. 4*A*).

For ischemic oxygen concentrations lower than 5% of physiological levels, model simulations predict that cellular ATP levels are depleted after approximately 400 s, similar to results reported by McDougal and Dewey (22) and Ch’en *et al.* (27). Below 5%, energy buffering enzymes such as creatine kinase (CK) and adenylate kinase (AK) initially keep ATP levels at normal physiological levels by using intracellular stores of creatine phosphate, glycogen, and ultimately ADP. Interestingly, the qualitative behavior of the recovery of ATP, *i.e.*, the temporal trajectory, seems to be the same, regardless of ischemic oxygen concentrations, while only the starting point of the ATP production is postponed with decreasing oxygen concentrations (see Fig. 4*A*).

After reintroducing preischemic oxygen levels, the ATP stores are already fully recovered after approximately 200 s of reperfusion, regardless of the severity of ischemia. Additionally, in Figure 4*B* we show how mitochondrial ATP production *via* oxidative phosphorylation quickly stops at the onset of ischemia due to a reduced activity of protein complexes and consequently a reduced proton-motive force. The anaerobic metabolism takes over the ATP production during the first minutes of ischemia. At approximately 350 s of ischemia ATP levels approach 0 for ischemic oxygen concentrations below 1%. Due to their importance in the scope of ROS production and subsequent oxidative damage, we also tracked the reaction rates at protein complex I and III of the electron transport chain. While they are tightly coupled, the results indicate that the reaction rate at complex III approaches 0 within the first seconds of ischemia, whereas complex I seems to be working at a greatly reduced activity throughout the period of ischemia (see Fig. 4, *B* and *D* respectively).

The model predicts that at ischemic oxygen concentrations above or equal to 5% of the normal physiological level, the overall reaction rate at complex I decreases instantly at the onset of ischemia and also recovers quickly at the onset of reperfusion. However, when reperfusion follows an ischemic period at even lower ischemic oxygen concentrations, the reaction rate at complex I becomes slightly negative before recovering to preischemic levels, indicating that complex I runs in reverse for a short period of time. Again, the simulations show highly nonlinear responses to different ischemic oxygen concentrations at the onset of reperfusion. Additionally, the reaction rate at complex III exhibits a large spike during the first seconds of reperfusion to levels approximately threefold of the preischemic values.

In Figure 4*C*, we plot the polarization of the mitochondrial membrane potential during ischemia and reperfusion. Being directly affected by the reactions at complex I and III, the membrane potential also decreases rapidly at the onset of ischemia before asymptotically approaching 55 mV. Upon reperfusion, the membrane potential spikes to a level twice the preischemic value for approximately 2 min before settling at preischemic levels. If ischemic oxygen concentrations stay at 5% or above, the membrane potential exhibits a less drastic decrease at the onset of ischemia, and upon reperfusion the potential does not overshoot. Thus, there appears to be a threshold of oxygen during ischemia that leads to hyperpolarization of the mitochondrial membrane upon reperfusion. Interestingly, the magnitude of the potential spike seems to be independent of the ischemic oxygen levels past a certain threshold, whereas the duration of the elevated membrane potential is inversely related to ischemic oxygen levels.

### A two-step reperfusion protocol

**Figure 5:**
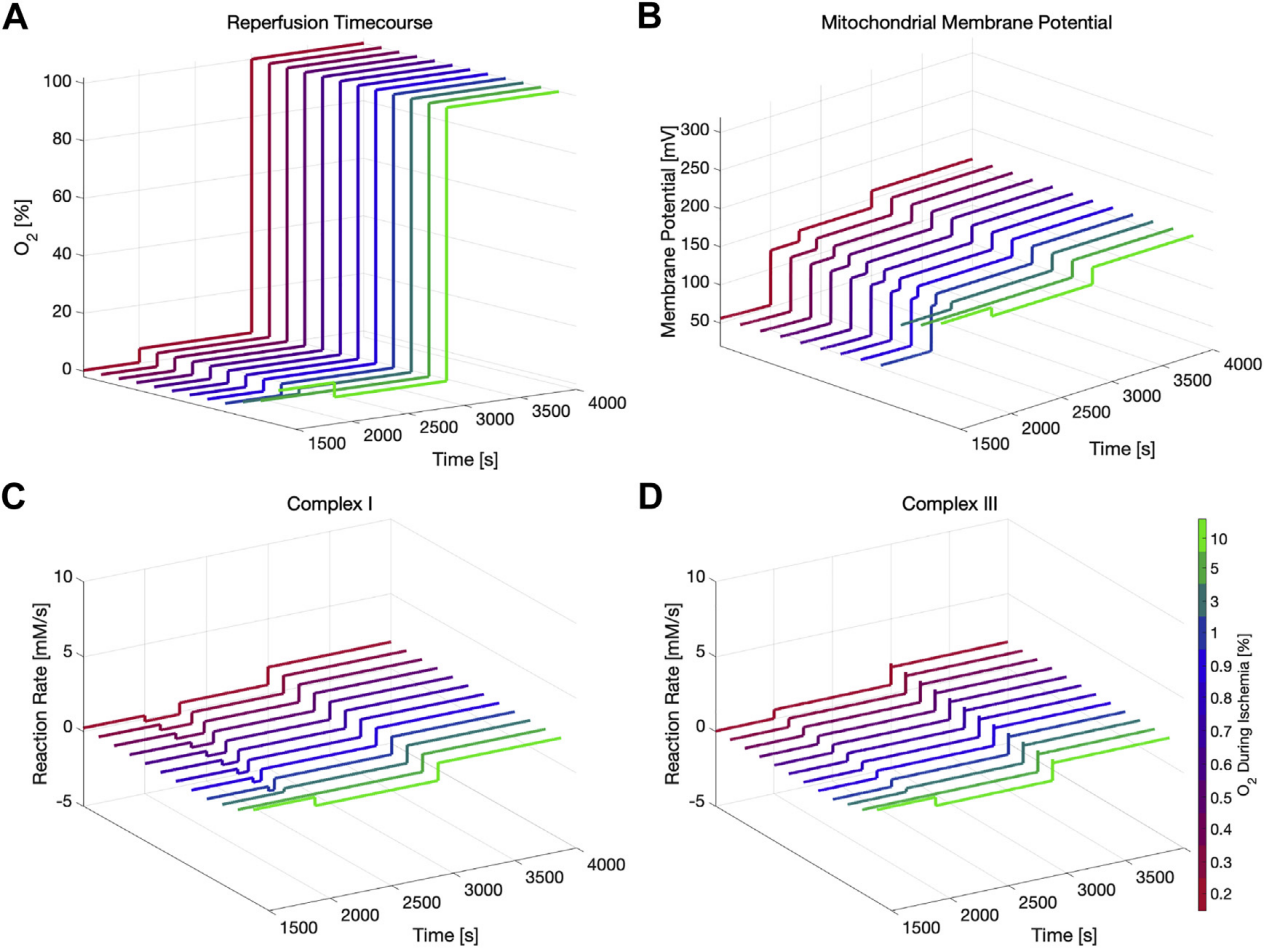
**Ischemia/reperfusion simulations for different levels of residual ischemic oxygen concentrations and a two-step reperfusion protocol.** Oxygen levels during ischemia ranged from 0.2% to 10% of physiological levels. At 2000 s, oxygen levels were adjusted to 5% of physiological levels (first step of reperfusion) and at 3000 s, oxygen levels were returned to physiological levels (second step of reperfusion). Traces of (*A*) oxygen concentrations, (*B*) mitochondrial membrane potential, (*C*) reaction rate at complex I, and (*D*) reaction rate at complex III are color-coded based on the initial level of ischemia.

**Figure 6:**
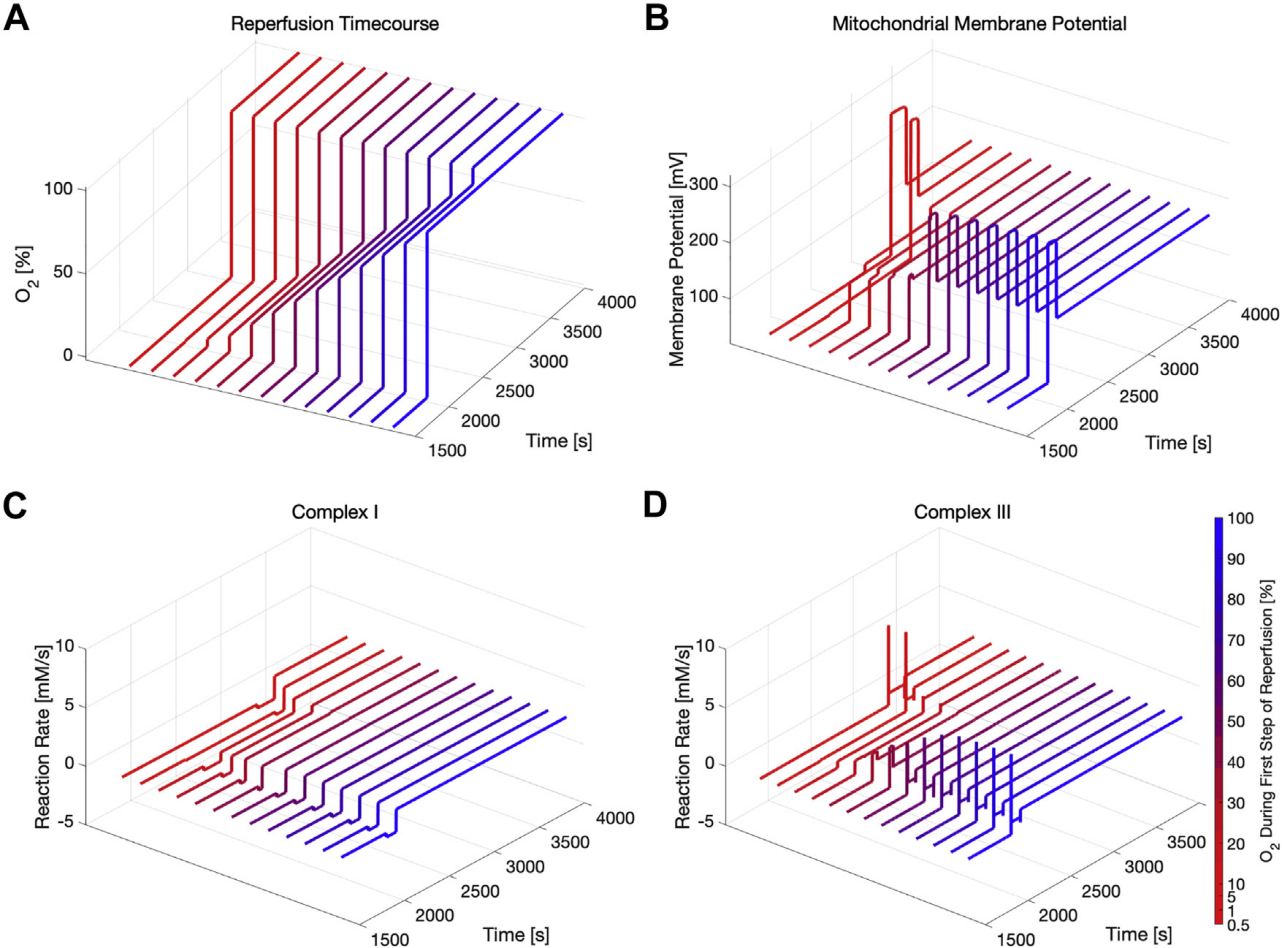
**Ischemia/reperfusion simulations for different levels of residual oxygen concentrations during the first step of a two-step reperfusion protocol.** In each simulation, the oxygen level during ischemia was maintained at 0.5% of physiological levels. At 2000 s, the oxygen level was adjusted to a concentration ranging from 1 to 100% of physiological levels (first step of reperfusion) and at 3000 s, oxygen levels were returned to physiological levels (second step of reperfusion). Traces of (*A*) oxygen concentrations, (*B*) mitochondrial membrane potential, (*C*) reaction rate at complex I, and (*D*) reaction rate at complex III are color-coded based on the oxygen level during the first step of reperfusion.

In order to reduce the sources of reperfusion injury, a two-step reperfusion protocol was simulated (see Figs. 5 and 6). First, ischemia was introduced for 1000 s. Instead of going back to physiological oxygen levels immediately after reperfusion, an intermediate step with oxygen levels not fully at 100% was added for another 1000 s. In Figure 5, the mentioned two-step reperfusion protocol is simulated for different ischemic oxygen concentrations and a constant first-step reperfusion oxygen concentration of 5%. Figure 5*B* shows that the dominant part of the increase in mitochondrial membrane potential is regained with even as small an oxygen level of reperfusion as 5% of physiological oxygen (*i.e.*, the membrane potential recovers already during the first reperfusion step, regardless of the ischemic oxygen values). The complex I negative reaction rate (reverse electron transport) shows up distinctly at the initiation of the first step of reperfusion, and its duration increases with decreasing ischemic oxygen concentrations (see Fig. 5*C*). Similar to Figures 5 and 6 shows the influence of the oxygen concentration during the first wave of reperfusion. Here, the ischemic oxygen concentration was set to 0.5% of physiological values and followed by various two-step reperfusion scenarios, with the first reperfusion step ranging from 1% to 100% of physiological O_2_ levels. The spike in mitochondrial membrane potential was suppressed for cases when 5% to 10% oxygen was utilized for the first reperfusion step (see Fig. 6*B*). Similarly, Figure 6*C* shows that reverse electron transport at complex I is minimized for the same range of oxygen levels; and lastly, Figure 6*D* indicates that this range of oxygen levels results in minimal spiking of the reaction rate at complex III. All of these observations suggest that there is an optimal range for the oxygen concentration during the first step of the reperfusion that minimizes ROS production and consequently reperfusion injury.

## Discussion

### Model validation

The model developed herein has been validated with experimental values obtained during normal physiological and pathological conditions and from a variety of species. Figure 1 shows that the extensions introduced to the McDougal model (22) result in similar steady-state values for cytosolic metabolites as measured in perfused rat hearts by Kashiwaya *et al.* (24). Additionally, model predictions for ischemia and reperfusion conditions correlate well with experimental values measured by Wu *et al.* (25) and Clarke *et al.* (26). However, the ATP/ADP ratio during reperfusion measured in guinea pig hearts could not be reproduced precisely (see Fig. 3*F*). Notably, in the work of Clarke *et al.* (26), this ratio was not directly measured but calculated using the creatine phosphate and pH measurements and assuming a constant creatine pool and creatine kinase equilibrium. Two possible reasons for the mismatch between model predictions and experimental results could be the following: (A) The difference in the total amount of creatine ([CrP] + [Cr]) results in different ATP/ADP dynamics during ischemia and reperfusion. Figure 3*D* shows that creatine phosphate is fully recovered after about 60 s according to the model, while the experimental values indicate that it only takes approx. 20 s. These different timescales can also be observed in Figure 3*F*. (B) The assumption of a constant cytosolic pH results in a too simplified model such that the ATP/ADP dynamics upon reperfusion cannot be captured correctly. However, in this context this is unlikely since Clarke *et al.* report that pH is the value with the slowest transition time during ischemia and reperfusion. It is also possible that the high collateral circulation present in guinea pig hearts results in a higher residual oxygen concentration than used in the simulations (Clarke *et al.* showed that there were no significant drops in ATP during 150 s of ischemia in guinea pig hearts). To verify this, simulations were also carried out with higher residual oxygen concentrations (up to 10 % of physiological levels); however, this did not result in an initial overshoot of the ATP/ADP ratio, as it can be seen in Figure 3*F*. Even though the characteristic overshoot of the ATP/ADP ratio seen in Figure 3*F* cannot be reproduced by the model, it accurately predicts that the ATP/ADP ratio reaches steady state after approx. 75 s. This is also consistent with the experimental data.

The model predictions were also compared with experimental measurements by Wu *et al.* (25), who used phosphate-magnetic resonance spectroscopy (P-MRS) to measure creatine phosphate concentrations in the left anterior descending artery in canine hearts under different cardiac work states. This comparison showed that predictions of creatine phosphate levels during ischemia and reperfusion correlate well with experimental values (see Fig. 2). Experimental and predicted data both show a close to instant response to reduction in cellular oxygen levels and use the creatine phosphate stores to keep ATP levels at preischemic values for as long as possible. Furthermore, these creatine phosphate stores are also refilled immediately, once sufficient oxygen is available to generate ATP through oxidative phosphorylation.

Sensitivity analysis at multiple timepoints shows how the control mechanism of the metabolic network changes between physiological and pathological conditions. During normal oxygen concentrations, most of the glycolytic metabolite levels are regulated by the enzymes hexokinase and phosphofructokinase. This behavior is consistent with other studies: indeed, hexokinase, phosphofructokinase, and pyruvate kinase are generally assumed to be the flux controlling enzymes of glycolysis. However, it has been shown that pyruvate kinase does not seem to limit the overall flux of glycolysis in hearts since the rate of pyruvate kinase is linked to the glycolytic flux (28, 29). Additionally, the concentrations of energy-related molecules such as adenosine and creatine are strongly regulated by the ATP consumption rate and the adenine nucleotide translocase (a transporter protein that exchanges ATP and ADP between cellular compartments).

During ischemic conditions, mitochondrial reactions included in the model do not exhibit any control since oxidative phosphorylation needs oxygen to produce ATP. Therefore, all reaction rates in the mitochondria related to oxidative phosphorylation asymptotically converge to 0. Surprisingly, however, the enzyme exerting the highest regulation of cytosolic concentrations is glucose-6-phosphate dehydrogenase, which acts as a sink for the glycolysis intermediate G6P. This is interesting because the pentose phosphate pathway is not included in this model. The pentose phosphate pathway is a metabolic pathway parallel to glycolysis that produces sugars to make DNA, RNA, and NADPH molecules used in, *e.g.*, fatty acid synthesis (30).

As expected, the sensitivity analysis performed after reperfusion shows similar results to preischemic conditions since the reaction parameters are not influenced by the ischemic period. This behavior arises due to the fact that there are no mechanisms for irreversibility or cellular damage included in the current model. An improved model version would potentially include a mechanism for cellular damage as a function of the time and severity of ischemia. It is common in ischemia/reperfusion simulations to change reaction parameters between pre- and postischemic periods (*e.g.*, Chouchani *et al.* (16) reduced the maximum reaction rate of ATP synthase to 50%, and Bazil *et al.* (31) increased the complex II activity and equilibrium constant by a factor of 10 after reperfusion). However, in many cases these parameter changes have only been qualitatively estimated, while in the current model, the focus was on determining the biochemical changes on a quantitative level.

### Oxygen dependence and oxidative damage

Results shown in Figure 4 suggest that cardiomyocytes are able to survive at oxygen concentrations as low as 10% of the normal physiological value. This is in agreement with results reported by McDougal and Dewey (22), who suggested a minimum viable oxygen concentration of 0.01 mM (8.5% of physiol. level) for nonbeating cardiomyocytes and 0.006 mM (4.6% of physiol. level) for cardiomyocytes that just keep up the most essential functions to survive. This is especially interesting, because McDougal and Dewey assumed a constant ATP consumption rate for the nonbeating and maintenance-level states of 56% and 21% of the healthy ATP consumption. In this work, we refined this assumption by including the dynamic ATP consumption rate suggested by Wu *et al.* (25). Wu *et al.* modeled ATP consumption as a function of the relative molecular concentrations of Pi, ADP, and ATP and validated the predictions in full-occlusion protocols on canine hearts. Additionally, the values were consistent with similar protocols on canine hearts, measured by Schwartz *et al.* (32). Therefore, the current model does not make any assumptions about the state of the cardiomyocyte (*i.e.*, beating, nonbeating, maintenance-only), but implicitly adjusts the ATP consumption and consequently creates a potential readout for the cardiomyocyte state based on metabolic measurements. However, the parameters in the equation associated with the ATP consumption rate are based on experiments with canine hearts. Therefore, further biological experiments using human cardiomyocytes will be necessary to assess whether these results are also applicable to humans.

During ischemia, the mitochondrial membrane potential asymptotically approaches a value of approximately 55 mV. Dissecting the equation for the change of the membrane potential over time into its components (see Table 2) shows that during prolonged ischemia, all chemical reaction rates approach 0 except for the proton leak. Thus, the membrane potential is only dissipated *via* the proton leak. This is also in agreement with Borutaite *et al.* (33), who measured the effects of the respiratory chain and the proton leak on the mitochondrial membrane potential in isolated mitochondria. The authors showed that during ischemia, the control coefficient for the proton leak increases while the coefficient for the respiratory chain decreases. It was further hypothesized that this was driven by an increase in permeability of the mitochondrial membrane to protons.

**Table 2:** Rate of change for species in mitochondrial intermembrane space

$\frac{\text{d}\left[\text{Species}\right]}{dt}\cdot V_{IMSpace}$	Type	Expression
${\left[\text{ADP}\right]}_{\text{i}}$	Assignment	${\left[\text{ADP}\right]}_{\text{x}}$
${\left[\text{fADP}\right]}_{\text{i}}$	ODE	$-J_{MgADPi}$
${\left[\text{mADP}\right]}_{\text{i}}$	ODE	$J_{MgADPi}$
${\left[\text{ATP}\right]}_{\text{i}}$	Assignment	${\left[\text{ATP}\right]}_{\text{x}}$
${\left[\text{fATP}\right]}_{\text{i}}$	ODE	$-J_{MgATPi}$
${\left[\text{mATP}\right]}_{\text{i}}$	ODE	$J_{MgATPi}$
${\left[\text{H}\right]}_{\text{i}}$	Const.	n.A.
${\left[\text{K}\right]}_{\text{i}}$	Const.	n.A.
${\left[\text{Pi}\right]}_{\text{i}}$	ODE	$J_{Pi2}-J_{Pi1}$
${\left[\text{Cox}\right]}_{\text{i}}$	ODE	$2J_{C4}-2J_{C3}$
${\left[\text{Cred}\right]}_{\text{i}}$	ODE	$2J_{C3}-2J_{C4}$
ΔΨ	ODE	$\left(4J_{C1}+2J_{C3}+4J_{C4}-3J_{F1}-J_{ANT}-J_{Hle}\right)/C_{IM}$∗

Type indicates the method used to calculate concentration levels. ODE: expression refers to rate of change $\frac{\text{d}\left[\text{Species}\right]}{dt}\cdot V_{IMSpace}$. Assignment: concentration is directly calculated based on expression. Const: Concentration is fixed.

∗ Except for ΔΨ, where the rate of change directly refers to $\frac{\text{dΔΨ}}{dt}$.

Although we have modeled only 15 min of ischemia, cardiomyocyte damage can occur during this short period. Early observations of cardiomyocytes revealed structural changes to the mitochondria after only 5 to 10 min of ischemia (34), and more recent studies have shown depletion of desmin, actin, myoglobin, along with the presence of fibrinogen and C5 in cardiomyocytes after only 15 min of ischemia. Early canine studies also indicated that short periods of ischemia (15–20 min) were accompanied by substantial accumulation of lactic acid, along with depletion of glycogen and ATP (35, 36). While these effects could be reversed quickly during reperfusion, cardiac function was significantly impacted and slow to recover after these short ischemic intervals, as indicated by a high incidence of ventricular fibrillation upon reperfusion (35), and persistently depressed contractile function even after 50 min of reperfusion (36). Furthermore, myocardial electrical impedance has been shown to increase in a biphasic manner after ischemia, exhibiting an early increase after onset of ischemia, a plateau phase, and a second rapid increase associated with cellular uncoupling (37). Myocardial electrical impedance reaches a plateau after 46 min of ischemia in dogs, but after less than 5 min of ischemia in humans, reflecting the observation that human myocardium is particularly sensitive to ischemia, even if it is of short duration (37).

With decreasing ischemic O_2_ values, the ATP pool starts to deplete at an increasing speed (see Fig. 4*A*). Additionally, the data in Figure 4 indicates that, at O_2_ levels below 10% of normal physiological levels, conditions favorable for the generation of reactive oxygen species are present. Below this threshold value, the model predicts a reversal of the electron transport at complex I and a spike in the mitochondrial membrane potential, which is associated with excessive superoxide production at complex I and III, respectively (16, 38, 39). Both of these phenomena could potentially be explained by the altered redox state of the electron transport chain. In physiological conditions, the individual protein complexes in the electron transport chain act as a supercomplex and their activity is highly coupled. This is also observed in model predictions, where it can be seen that the reaction rates at complex I and III are highly correlated during the preischemic and ischemic period. However, this correlation is not observed anymore during reperfusion, as shown in Figure 4, *B* and *D*. During ischemia, the sequential stopping of complex IV, III, and then I leads to fully reduced coenzyme Q and cytochrome C pools. Upon reperfusion, the cytochrome C pool rapidly drives the proton pumping of complex IV leading to a spike in the membrane potential. Simultaneously, the redox driving force resulting from the reduced state of coenzyme Q leads to a reversal of the chemical reaction at complex I and therefore reverse electron transport (RET). The chemical flux through complex I is represented as a thermodynamically balanced model and is driven toward a steady state, which is a function of the proton gradient and the mitochondrial membrane potential. A reduced coenzyme Q pool during ischemia combined with a high mitochondrial membrane potential thus drives the chemical reaction at complex I to reverse and as a consequence to a reverse transfer of electrons from ubiquinol to complex I. This has also been discussed by Scialò *et al.* (40), who reviewed different mechanisms of ROS production at complex I during forward and reverse electron transport.

While the elevation of complex III activity (together with complex IV) is quite short-lived, the hyperpolarization during reperfusion negatively correlates with activity of the mitochondrial proton leak, suggesting that increased activity of the proton leak may impact the mitochondrial membrane potential. This has also been discussed by Cheng *et al.* (41), who mentioned the existence of a protective feedback loop between ROS generation and proton leak activity. Based on our model results, we hypothesize that this feedback loop may be involved in the membrane potential observed during reperfusion.

Chouchani *et al.* recently proposed that RET is a key driver of oxidative damage during reperfusion (16, 42). Our model predicts a drastically increased mitochondrial membrane potential during the first minutes of reperfusion. A potential explanation for this could be the large redox driving force that pushes protons across the mitochondrial membrane to establish the electrochemical gradient used for ATP production. During ischemia, the coenzyme Q and cytochrome C pools become fully reduced. Thus, at the first moments of reperfusion, reactions at complex I, III, and IV will have a shifted chemical equilibrium resulting in faster reaction rates and accordingly more protons being pumped across the membrane simultaneously. It has been shown that an increased mitochondrial membrane potential can lead to a strong increase in superoxide production at complex III (38, 39). A high membrane potential slows down the electron transfer from heme bL to heme bH (43), two subunits of complex III. Therefore, the predicted spike of the membrane potential seems to lead to favorable conditions for superoxide production at complex III (44). In isolated mitochondria under state 4 conditions and succinate (supporting RET), mitochondrial membrane potential increases of 30 to 50 mV (to nearly 200 mV) have been observed (45). Although the absolute value of dPsi predicted is higher than that seen in biological experiments, the scenario of high dPsi, maximal NADH pool, and reverse electron transport has been previously described (45, 46). However, even though superoxide production due to the increase in membrane potential has been discussed in different studies (*e.g.*, (47)), few papers have been discussing the mechanism of superoxide production as a result of the mitochondrial membrane potential in the context of ischemia/reperfusion injury. It is therefore critical to design appropriate biological experiments that are able to confirm whether this identified mechanism is a driver of oxidative damage in the pathophysiology of reperfusion injury.

### A strategy to minimize ischemia/reperfusion injury

Simulations of the stepwise reperfusion shown in Figures 5 and 6 suggest that biochemical modeling could help in identifying optimal reperfusion strategies to minimize tissue damage from reperfusion injury in clinical applications. Figure 5 indicates a correlation between tissue damage and severity of ischemia (*i.e.*, recovery time for the mitochondrial membrane potential and time of reverse electron transport at complex I increase with lower oxygen concentrations during ischemia). Interestingly, however, the spiking of the membrane potential and the complex III reaction rate shown in Figure 4, *C* and *D* disappear when introducing the intermediate reperfusion step at 5% oxygen (see Fig. 5, *B* and *D*), suggesting an improved reperfusion outcome. In addition to the investigation of the relationship between tissue damage and severity of ischemia during the two-step reperfusion shown in Figures 5 and 6 indicates different degrees of oxidative damage as a function of the chosen reperfusion strategy. Thus, while Figure 5 is important from a biological perspective, Figure 6 is especially interesting from a clinical perspective as it directly suggests potential intervention strategies in a clinical setting. The mitochondrial membrane potential and complex I and III reaction rate shown in Figure 6 consistently indicate reduced damage from reperfusion injury when introducing an intermediate reperfusion step at optimized oxygen levels. The spike in the mitochondrial membrane potential (and the associated ROS production) is minimized when the oxygen concentration of the first reperfusion step is between 5% and 10% (see Fig. 6*B*, traces 3 and 4 from left to right). Similarly, Figure 6, *C* and *D* show a minimal RET at complex I and the spike in the reaction rate of complex III nearly disappeared when introducing this intermediate reperfusion step. These results are also supported by experimental findings of different groups (48, 49, 50). Abdel-Rahman *et al.* (51) showed in a porcine model and in a clinical study of 19 patients undergoing cardiac surgery (48) that a graded reperfusion led to a decrease in myocardial oxidative injury. In a recent review of hypoxemic reperfusion, Tasoulis *et al.* (52) mention that a stepwise reperfusion reduces the available oxygen for ROS production, while still providing enough oxygen for the cell to recover from ischemia. Thus, the initial burst in ROS generation could be potentially mitigated by a gradual reperfusion. This indicates that (a) a multistep reperfusion strategy could outperform a one-step reperfusion regardless of ischemic oxygen concentrations and (b) a mathematical model of the cardiomyocyte metabolism, such as the one developed herein, could be used to identify optimal reperfusion strategies for clinical settings. To the best of our knowledge, the changes in molecular-level species during stepwise reperfusion and the associated biological mechanisms have not been studied extensively *in vivo* yet. Thus, it will be critical to design and perform adequate experiments that validate these findings and additionally to come up with an according theory that is able to explain the mechanisms responsible for the potentially beneficial effect of a stepwise reperfusion protocol.

For the future, it will be important to refine the model in these directions to quantify the exact amounts of ROS being produced at the protein complexes of the electron transport chain and follow up these predictions with biological experiments. Gauthier *et al.* (53) created such a computational model of ROS dynamics in cardiac mitochondria and suggest that ROS production increases exponentially as a function of the mitochondrial membrane potential. This further highlights the importance of our hypothesis that the membrane potential seems to be a key mechanism in ischemia/reperfusion injury.

It is important to note that, under certain circumstances, ROS can also exert cardioprotective mechanisms by activating HIF-1 (13, 54) and Nrf2 (55) signaling pathways. In fact, the cardioprotective effects of ischemic preconditioning are mediated in part by activation of HIF-1, which inhibits mPTP opening (56), and Nrf2 signaling, which increases activation and expression of antioxidant enzymes (57, 58). Additionally, postconditioning approaches, which bear some similarity to the two-step reperfusion protocol that we implement in our model, elicit cardioprotection through various mechanism that include attenuation of oxidative stress and inhibition of mPTP opening (59, 60, 61, 62, 63). Although many studies have indicated that postconditioning reduces ROS production at reperfusion (64, 65), others have demonstrated that ROS scavenging attenuates the effects of postconditioning (66, 67, 68). Whether ROS acts as a mediator of injury or protection seems to depend critically on the amount and biological context of ROS production, with high or increased levels being associated with deleterious (13, 42, 69) and low or minimal levels being associated with protective (70) effects. Although we do not directly model ROS generation in our system, we believe that the large change in dPsi during reperfusion would generate relatively high levels of ROS, since ROS production at complex III has exponential dependence on dPsi (44, 71). We therefore hypothesize that the resultant high levels of ROS would exert a deleterious, rather than protective, effect on the myocyte.

The model could be extended to include other energy-related pathways such as fatty acid oxidation and processes related to muscular contractions including calcium dynamics and more general electrophysiological mechanisms (72, 73). With our model being primarily focused on glucose as a substrate, this could be an excellent addition to study the role of additional metabolic substrates within the scope of ischemia/reperfusion injury.

Yaniv *et al.* (74) highlighted ADP/Pi and calcium as main regulators of ATP production and consumption in the mammalian heart. While the ADP and Pi controlled ATP consumption rate was included in our work, we do not have a direct relationship between calcium and ATP consumption. Calcium at least partially controls mitochondrial ATP production by regulating the activation of various enzymes in the Krebs cycle and the electrochemical gradient established in the electron transport chain. In order to capture these mechanisms, the phenomenological representation of the Krebs cycle used in this model could be replaced by including the chemical kinetics of the individual dehydrogenases, such as done by Wu *et al.* (75) in their mathematical representation of the Krebs cycle.

Our model does not yet incorporate mechanisms that directly tie changes in mitochondrial injury during reperfusion to cell death mechanisms. It has been shown that excessive ROS production, rapid pH restoration, and intracellular calcium overload during reperfusion (7) can lead to disruption of mitochondrial integrity through the opening of mitochondrial permeability transition pore (MPTP) (76, 77). MPTP opening, in turn, can cause mitochondrial swelling and rupture, releasing intermembrane proteins such as cytochrome c and apoptosis-inducing factor that activate cell death pathways (78). Several types of cell death, including apoptosis and necrosis, are implicated in I/R injury (79, 80), and future work should focus on expanding our computational framework to model these mechanisms.

In the scope of this work, only the temporal dynamics of the cardiac metabolism were incorporated. However, the concentration of many species and organelles such as the mitochondria are not spatially homogeneous. Balaban *et al.* (81) showed that cardiac mitochondria are organized in regional subnetworks, which was suggested to limit the spread of mitochondrial dysfunction to local regions of the cell. In addition, the model could be extended to include the time delays inherent in reperfusion dynamics and diffusion of oxygen from the vasculature to the cardiomyocytes, as both effects would influence the time course of oxygen delivery to the reperfused cardiac tissues. To include such mechanisms, an improved simulation of the cardiac metabolism would be based on a spatiotemporal model.

However, the focus of this work was to develop an experimentally validated model of the cardiomyocyte metabolism to gain insights into the complex processes and reactions present during ischemia/reperfusion injury. Additionally, we aim to provide the research community with a metabolic model that can be easily extended based on a specific research question, such as ROS production, cardiac muscle contractions, or fatty acid oxidation. To achieve this, the herein developed model is available online on http://www.ebi.ac.uk/biomodels/. Modeling cellular conditions though ischemia and reperfusion will enable researchers to study the mechanisms of reperfusion injury in cardiomyocytes *in silico* and also help to plan and conduct biological experiments in order to improve the understanding of the pathophysiology of ischemia/reperfusion injury.

In summary, we have implemented a model of the cardiomyocyte metabolism, which is able to simulate the pathophysiological states of ischemia/reperfusion injury as a function of different extracellular oxygen levels. Additionally, the model was qualitatively and quantitatively validated using experimental measurements several animal species. The model suggests that cardiomyocytes are capable of adjusting their energy production in response to drastically varying extracellular oxygen availabilities. More specifically, the results show that oxygen concentrations as low as 10% of the normal physiological levels are enough for the cells to survive. Our results suggest that changes in the redox state of the electron transport chain are likely responsible for oxidative damage occurring during reperfusion injury. Reverse electron transport and a highly increased mitochondrial membrane potential both create conditions for increased rates of ROS production and thus oxidative damage. Most importantly, the model shows potential intervention strategies such as a two-step reperfusion protocol, in order to minimize oxidative damage during the first moments of reperfusion.

## Experimental procedures

To track the time course of ATP and other related species, a model comprised of five compartments and 67 species was created using a systems biology approach. This resulted in a nonlinear system of ordinary differential equations (ODEs). This implementation builds upon the model McDougal and Dewey (22), describing the anaerobic metabolism in cardiomyocytes. Their model is mostly focused on the cytosolic part of the energy production, *i.e.*, glycolysis, glycogenolysis, and glycogenesis and cytosolic ATP buffering. In this work, this description was extended by incorporating a more complete and exact model of the mitochondrial ATP production inspired by the work of Beard (23). Additionally, the constant ATP consumption rate was replaced by a phosphate and ADP-dependent rate as suggested by Wu *et al.* (25). Generally speaking, the model starts at the diffusion of oxygen from the blood vessel into the extracellular space. From there, glucose and oxygen diffuse through the cellular membrane into the cytosol of the cardiomyocyte. Glucose then enters the glycolytic pathway with pyruvate being the final metabolite. In our model, we integrated the conversion of pyruvate into acetyl-coenzyme A and the subsequent citric acid cycle into a single phenomenological process that reduces NAD to NADH, as suggested by Beard (23). This NADH then enters the electron transport chain at complex I to transfer electrons to complex IV with oxygen being the final electron acceptor. Simultaneously these protein complexes transfer protons from the mitochondrial matrix into the intermembrane space. The resulting proton-motive force, consisting of the proton gradient and the mitochondrial membrane potential, is finally used by the F1F0-ATP synthase to produce ATP from ADP and inorganic phosphate.

To formulate a system of ODEs representing the dynamics of the metabolic processes, the reactions involving this species were listed for every species represented in the model. Afterward, the flux of each reaction and/or diffusion process was calculated based on either Michaelis–Menten kinetics or diffusion equations. Finally, the rate of change for each species was calculated as the sum of the reactions producing this species minus the sum of reactions consuming the species plus (or minus) the reactions/diffusion processes that transport a species in or out of its compartment and represented by a system of nonlinear ordinary differential equations. This is illustrated in the following equation

$$\frac{\text{d}\left[{\text{C}}_{\text{i}}\right]}{\text{dt}}=J_{Production}\pm J_{Transport}-J_{Consumption}$$

where $\left[{\text{C}}_{\text{i}}\right]$ refers to the concentration of species *i* in mM, $J_{Production}$ and $J_{Consumption}$ represent the amount of $\left[{\text{C}}_{\text{i}}\right]$ that is being produced and consumed respectively and $J_{Transport}$ is the amount of species *i* that is transported in and out of the compartment per unit time. For a full list of the model equations and initial values, see the corresponding tables in the following section.

### Compartments

#### Cytosol

Most species in the cytosol are: (1) associated to glycolysis; (2) forms of adenosine phosphate; (3) related buffer systems. All kinetic parameters related to processes in the cytosol are taken from McDougal and Dewey (22), except transport processes between the cytosol and the mitochondria. See Table 3 for species and initial conditions and Table 4 for the rate of change for each species.

**Table 3:** Cytosolic species

Species	Full name	Unit	Initial value	Source
${\left[\text{AMP}\right]}_{\text{c}}$	Adenosine Monophosphate	mM	$1\times 10^{-5}$	(27)
${\left[\text{ADP}\right]}_{\text{c}}$	Adenosine Diphosphate	mM	$1\times 10^{-5}$	(27)
${\left[\text{ATP}\right]}_{\text{c}}$	Adenosine Triphosphate	mM	7	(27)
${\left[\text{NAD}\right]}_{\text{c}}$	Nicotinamide Adenine Dinucleotide (ox)	mM	2.2565	(22)
${\left[\text{NADH}\right]}_{\text{c}}$	Nicotinamide Adenine Dinucleotide (red)	mM	0.7135	(22)
${\left[\text{Glucose}\right]}_{\text{c}}$	Glucose	mM	1.91	(84)
${\left[13\text{BPG}\right]}_{\text{c}}$	1,3-Bisphosphoglycerate	mM	$8.69\times 10^{-4}$	(24)
${\left[2\text{PG}\right]}_{\text{c}}$	2-Phosphoglycerate	mM	0.009	(24)
${\left[3\text{PG}\right]}_{\text{c}}$	3-Phosphoglycerate	mM	0.071	(24)
${\left[\text{Cr}\right]}_{\text{c}}$	Creatine	mM	0	(27)
${\left[\text{CrP}\right]}_{\text{c}}$	Creatine Phosphate	mM	25	(27)
${\left[\text{DHAP}\right]}_{\text{c}}$	Dihydroxyacetone Phosphate	mM	0.036	(24)
${\left[\text{F}16\text{BP}\right]}_{\text{c}}$	Fructose 1,6-Bisphosphate	mM	$6.78\times 10^{-4}$	(24)
${\left[\text{F}6\text{P}\right]}_{\text{c}}$	Fructose-6-Phosphate	mM	0.041	(24)
${\left[\text{G}16\text{BP}\right]}_{\text{c}}$	Glucose-1,6-Bisphosphate	mM	0.007	(24)
${\left[\text{G}1\text{P}\right]}_{\text{c}}$	Glucose-1-Phosphate	mM	0.02	(24)
${\left[\text{G}6\text{P}\right]}_{\text{c}}$	Glucose-6-Phosphate	mM	0.169	(24)
${\left[\text{GAP}\right]}_{\text{c}}$	Glyceraldehyde-3-Phosphate	mM	0.00162	(24)
${\left[\text{Glycogen}\right]}_{\text{c}}$	Glycogen	mM	21.4	(24)
${\left[{\text{L}}_{\text{i}}\right]}_{\text{c}}$	Lactate	mM	0.247	(22)
${\left[\text{LA}\right]}_{\text{c}}$	Lactic Acid	mM	155.84	(22)
${\left[\text{Mb}\right]}_{\text{c}}$	Myoglobin	mM	0.00543	(22)
${\left[{\text{MbO}}_{2}\right]}_{\text{c}}$	Saturated Myoglobin	mM	0.18457	(22)
${\left[{\text{O}}_{2}\right]}_{\text{c}}$	Oxygen	mM	0.11017	(22)
${\left[\text{PEP}\right]}_{\text{c}}$	Phosphoenolpyruvate	mM	0.013	(24)
${\left[\text{pH}\right]}_{\text{c}}$	pH	mM	7.1	(88)
${\left[\text{Pi}\right]}_{\text{c}}$	Inorganic Phosphate	mM	7	(27)
${\left[\text{PYR}\right]}_{\text{c}}$	Pyruvate	mM	0.055	(24)
${\left[\text{UDPG}\right]}_{\text{c}}$	Uridine Diphosphate Glucose	mM	0.099	(24)
${\left[\text{H}\right]}_{\text{c}}$	Hydrogen	mM	$7.94\times 10^{-4}$	(88)
${\left[\text{K}\right]}_{\text{c}}$	Potassium	mM	150	(23)
${\left[\text{Mg}\right]}_{\text{c}}$	Magnesium	mM	5	(23)
${\left[\text{mADP}\right]}_{\text{c}}$	Magnesium-bound ADP	mM	$9.35\times 10^{-6}$	(23)

**Table 4:** Rate of change for cytosolic species

$\frac{\text{d}\left[\text{Species}\right]}{\text{dt}}\cdot V_{cytosol}$	Type	Expression
${\left[\text{AMP}\right]}_{\text{c}}$	ODE	$J_{AK}$
${\left[\text{ADP}\right]}_{\text{c}}$	ODE	$-J_{CK}-2J_{AK}+J_{HK}+J_{PFK}-J_{PGK}-J_{PK}+J_{ATPase}-\alpha_{F1}J_{F1}$
${\left[\text{ATP}\right]}_{\text{c}}$	ODE	$J_{CK}+J_{AK}-J_{ATPase}-J_{HK}-J_{PFK}+J_{PGK}+J_{PK}+\alpha_{F1}J_{F1}$
${\left[\text{NAD}\right]}_{\text{c}}$	Const.	n.A.
${\left[\text{NADH}\right]}_{\text{c}}$	Const.	n.A.
${\left[\text{Glucose}\right]}_{\text{c}}$	Const.	n.A.
${\left[13\text{BPG}\right]}_{\text{c}}$	ODE	$J_{GAPDH}-J_{PGK}-J_{G16BPS}$
${\left[2\text{PG}\right]}_{\text{c}}$	ODE	$J_{PGM}-J_{Enolase}$
${\left[3\text{PG}\right]}_{\text{c}}$	ODE	$J_{PGK}-J_{PGM}+J_{G16BPS}$
${\left[\text{Cr}\right]}_{\text{c}}$	ODE	$J_{CK}$
${\left[\text{CrP}\right]}_{\text{c}}$	ODE	$-J_{CK}$
${\left[\text{DHAP}\right]}_{\text{c}}$	ODE	$J_{FBPA}-J_{TPI}-J_{G3PDH}$
${\left[\text{F}16\text{BP}\right]}_{\text{c}}$	ODE	$J_{PFK}-J_{FBPA}$
${\left[\text{F}6\text{P}\right]}_{\text{c}}$	ODE	$J_{PGI}-J_{PFK}$
${\left[\text{G}16\text{BP}\right]}_{\text{c}}$	Const.	n.A.
${\left[\text{G}1\text{P}\right]}_{\text{c}}$	ODE	$-J_{PGluM}-J_{UDPGP}+J_{GP}-J_{G16BPS}$
${\left[\text{G}6\text{P}\right]}_{\text{c}}$	ODE	$J_{HK}-J_{PGI}+J_{PGluM}-J_{G6PDH}$
${\left[\text{GAP}\right]}_{\text{c}}$	ODE	$J_{FBPA}+J_{TPI}-J_{GAPDH}$
${\left[\text{Glycogen}\right]}_{\text{c}}$	ODE	$J_{GSD}+J_{GSI}-J_{GP}$
${\left[{\text{L}}_{\text{i}}\right]}_{\text{c}}$	ODE	$J_{LF_{i}}-J_{LF_{o}}-J_{LA}+J_{LDH}$
${\left[\text{LA}\right]}_{\text{c}}$	ODE	$J_{LA}$
${\left[\text{Mb}\right]}_{\text{c}}$	ODE	$-J_{O_{2}Mb}$
${\left[{\text{MbO}}_{2}\right]}_{\text{c}}$	ODE	$J_{O_{2}Mb}$
${\left[{\text{O}}_{2}\right]}_{\text{c}}$	ODE	$-J_{C4}+J_{O_{2},EC}-J_{O_{2}Mb}$
${\left[\text{PEP}\right]}_{\text{c}}$	ODE	$J_{Enolase}-J_{PK}$
${\left[\text{Pi}\right]}_{\text{c}}$	ODE	$-J_{GAPDH}+2J_{UDPGP}-J_{GP}+J_{G6PDH}+J_{G3PDH}+2J_{G16BPS}+J_{ATPase}-\alpha_{F1}J_{F1}$
${\left[\text{PYR}\right]}_{\text{c}}$	ODE	$J_{PK}-J_{LDH}-\alpha_{DH}J_{DH}$
${\left[\text{UDPG}\right]}_{\text{c}}$	ODE	$J_{UDPGP}-J_{GSD}-J_{GSI}$
${\left[\text{H}\right]}_{\text{c}}$	Const.	n.A.
${\left[\text{K}\right]}_{\text{c}}$	Const.	n.A.
${\left[\text{Mg}\right]}_{\text{c}}$	Assignment	${\left[\text{Mg}\right]}_{\text{c},\text{total}}-{\left[\text{mADP}\right]}_{\text{c}}$
${\left[\text{mADP}\right]}_{\text{c}}$	Assignment	$\beta_{ADP}$

Type indicates the method used to calculate concentration levels. ODE: expression refers to rate of change $\frac{\text{d}\left[\text{Species}\right]}{\text{dt}}\cdot V_{cytosol}$. Assignment: concentration is directly calculated based on expression. Const: Concentration is fixed.

#### Mitochondrial intermembrane space

The intermembrane space acts mostly as a storage to sustain the proton gradient and the mitochondrial membrane potential necessary for the F1F0-ATP synthase to produce ATP from ADP and inorganic phosphate. Furthermore, since the F1F0-ATP synthase requires ADP and magnesium as a cofactor, binding between magnesium ions and ATP and ADP is also included in the intermembrane space and the mitochondrial matrix. Here, ADP and ATP always refer to the sum of magnesium bound (mADP, mATP) and free species (fADP, fATP). See Tables 2 and 5 for all species and corresponding rate of change equations in the intermembrane space.

**Table 5:** Species in mitochondrial intermembrane space

Species	Full name	Unit	Initial value	Source
${\left[\text{ADP}\right]}_{\text{i}}$	Adenosine Diphosphate	mM	10	(23)
${\left[\text{fADP}\right]}_{\text{i}}$	free ADP	mM	10	(23)
${\left[\text{mADP}\right]}_{\text{i}}$	Magnesium-bound ADP	mM	0	(23)
${\left[\text{ATP}\right]}_{\text{i}}$	Adenosine Triphosphate	mM	7	(23)
${\left[\text{fATP}\right]}_{\text{i}}$	free ATP	mM	7	(23)
${\left[\text{mATP}\right]}_{\text{i}}$	Magnesium-bound ATP	mM	0	(23)
${\left[\text{H}\right]}_{\text{i}}$	Hydrogen	mM	$7.94\times 10^{-5}$	(23)
${\left[\text{K}\right]}_{\text{i}}$	Potassium	mM	150	(23)
${\left[\text{Pi}\right]}_{\text{i}}$	Inorganic Phosphate	mM	1	(23)
${\left[\text{Cox}\right]}_{\text{i}}$	Cytochrome C (ox)	mM	1.7	(23)
${\left[\text{Cred}\right]}_{\text{i}}$	Cytochrome C (red)	mM	1	(23)
ΔΨ	Mitochondrial Membrane Potential	mV	170	(23)

#### Mitochondrial matrix

All processes in the mitochondrial matrix are either related to oxidative phosphorylation or transport processes between the mitochondrial matrix and the intermembrane space. First, pyruvate enters the mitochondrial matrix. Here it is worth mentioning that in this model the conversion of pyruvate into acetyl-coenzyme A and the TCA cycle are modeled as a single phenomenological dehydrogenase flux converting NAD into NADH, as suggested by Beard (23). This NADH is then used by the first protein complex (CI) in the electron transport chain to pump ions across the mitochondrial membrane. Similarly complex III and IV are pumping ions across the membrane to establish a proton-motive force, which is finally used by the F1F0-ATP synthase to produce ATP from ADP and inorganic phosphate. Although we have not modeled complex II explicitly, we incorporate the behavior of this complex implicitly *via* reactions occurring at complex I and III *via* changes in the Q and QH2 pools. For an overview of the species and rate of change equations in the mitochondrial matrix, refer to Tables 6 and 7, respectively.

**Table 6:** Species in mitochondrial matrix

Species	Full name	Unit	Initial value	Source
${\left[\text{ADP}\right]}_{\text{m}}$	Adenosine Diphosphate	mM	10	(23)
${\left[\text{fADP}\right]}_{\text{m}}$	free ADP	mM	10	(23)
${\left[\text{mADP}\right]}_{\text{m}}$	Magnesium-bound ADP	mM	0	(23)
${\left[\text{ATP}\right]}_{\text{m}}$	Adenosine Triphosphate	mM	7	(23)
${\left[\text{fATP}\right]}_{\text{m}}$	free ATP	mM	7	(23)
${\left[\text{mATP}\right]}_{\text{m}}$	Magnesium-bound ATP	mM	0	(23)
${\left[\text{H}\right]}_{\text{m}}$	Hydrogen	mM	$6.31\times 10^{-5}$	(23)
${\left[\text{K}\right]}_{\text{m}}$	Potassium	mM	140	(23)
${\left[\text{Mg}\right]}_{\text{m}}$	Magnesium	mM	5	(23)
${\left[\text{NAD}\right]}_{\text{m}}$	Nicotinamide Adenine Dinucleotide (ox)	mM	1.47	(23)
${\left[\text{NADH}\right]}_{\text{m}}$	Nicotinamide Adenine Dinucleotide (red)	mM	1.5	(23)
${\left[\text{Pi}\right]}_{\text{m}}$	Inorganic Phosphate	mM	1	(23)
${\left[\text{Q}\right]}_{\text{m}}$	Ubiquinone	mM	0.55	(23)
${\left[{\text{QH}}_{2}\right]}_{\text{m}}$	Ubiquinol	mM	0.8	(23)

**Table 7:** Rate of change for species in mitochondrial matrix

$\frac{\text{d}\left[\text{Species}\right]}{dt}\cdot V_{Mitochondria}$	Type	Expression
${\left[\text{ADP}\right]}_{\text{m}}$	ODE	$J_{ANT}-J_{F1}$
${\left[\text{fADP}\right]}_{\text{m}}$	ODE	$-J_{MgADPx}$
${\left[\text{mADP}\right]}_{\text{m}}$	ODE	$J_{MgADPx}$
${\left[\text{ATP}\right]}_{\text{m}}$	ODE	$J_{F1}-J_{ANT}$
${\left[\text{fATP}\right]}_{\text{m}}$	ODE	$-J_{MgATPx}$
${\left[\text{mATP}\right]}_{\text{m}}$	ODE	$J_{MgATPx}$
${\left[\text{H}\right]}_{\text{m}}$	ODE	$\frac{1}{r_{buff}}\cdot {\left[\text{H}\right]}_{\text{m}}\cdot \left(J_{DH}-5J_{C1}-2J_{C3}-4J_{C4}+2J_{F1}+2J_{Pi1}+J_{Hle}-J_{KH}\right)$
${\left[\text{K}\right]}_{\text{m}}$	ODE	$J_{KH}$
${\left[\text{Mg}\right]}_{\text{m}}$	ODE	$J_{MgADPx}-J_{MgATPx}$
${\left[\text{NAD}\right]}_{\text{m}}$	ODE	$J_{C1}-J_{DH}$
${\left[\text{NADH}\right]}_{\text{m}}$	ODE	$J_{DH}-J_{C1}$
${\left[\text{Pi}\right]}_{\text{m}}$	ODE	$J_{Pi1}-J_{F1}$
${\left[\text{Q}\right]}_{\text{m}}$	ODE	$J_{C3}-J_{C1}$
${\left[{\text{QH}}_{2}\right]}_{\text{m}}$	ODE	$J_{C1}-J_{C3}$

#### Extracellular space and blood vessel

Species present in the extracellular space and the blood vessel and their rates of change are listed in Tables 8 and 9. Additionally, all of the model compartments and their relative sizes are provided in Table 10.

**Table 8:** Species in extracellular space and vessel

Species	Compartment	Full name	Unit	Initial value	Source
${\left[{\text{O}}_{2}\right]}_{\text{e}}$	Extracellular Space	Oxygen	mM	0.1289	(22)
${\left[{\text{O}}_{2}\right]}_{\text{v}}$	Vessel	Oxygen	mM	0.1326	(22)
${\left[\text{L}\right]}_{\text{e}}$	Extracellular Space	Lactate	mM	0.33	(88)

**Table 9:** Species in extracellular space and vessel

$\frac{\text{d}\left[\text{Species}\right]}{dt}$	Type	Expression
${\left[{\text{O}}_{2}\right]}_{\text{e}}$	ODE	$\frac{{\text{J}}_{{\text{O}}_{2},\text{VE}}-{\text{J}}_{{\text{O}}_{2},\text{EC}}}{V_{extraceullular}}$
${\left[{\text{O}}_{2}\right]}_{\text{v}}$	Const.	n.A.

**Table 10:** Compartments involved in metabolic model

Compartment	Size	Unit	Source
Vessel	0.06842	l	(24)
Extracellular Space	0.24063	l	(24)
Cytosol	1	l	(24)
Mitochondrial Intermembrane Space	0.0715	l	(23)
Mitochondrial Matrix	0.6435	l	(23)

### Simulations

#### Time course

Time course simulations were generally performed for the duration of 3000 s, where the first 1000 s corresponded to normal physiological conditions of the cell, the time between 1000 s and 2000 s represented ischemia by decreasing the vascular oxygen levels from 0.133 mM to values between 10 and 0.1% of the physiological levels and after 2000 s reperfusion was simulated by switching the oxygen levels in the blood vessel back to preischemic values.

#### Sensitivity analysis

To analyze the sensitivity of the concentration of each species with respect to the rate of each individual chemical reaction or transport process, the sensitivity coefficients were computed as follows:

$$s_{ij}=\frac{\text{d}\left[{\text{C}}_{\text{i}}\right]}{{\text{dX}}_{\text{j}}}$$

where [C_i_] is the concentration of species *i* and X_j_ is the activity of the enzyme in reaction *j*. Furthermore, to work with unitless sensitivity coefficients, which allow for easier comparison between variables of different units (*e.g.*, membrane potential and ATP concentration), the relative sensitivity coefficients ${\tilde{s}}_{ij}$ were calculated as

$${\tilde{s}}_{ij}=s_{ij}\cdot \frac{{\text{X}}_{\text{j}}}{\left[{\text{C}}_{\text{i}}\right]}=\frac{\text{d}\left[{\text{C}}_{\text{i}}\right]}{{\text{dX}}_{\text{j}}}\cdot \frac{{\text{X}}_{\text{j}}}{\left[{\text{C}}_{\text{i}}\right]}$$

In order to characterize the total influence of an enzyme on the molecular concentrations, the L1-norm of the relative sensitivities across all species was calculated as

$${\tilde{S}}_{j}=\underset{i\in Species}{\sum }|{\tilde{s}}_{ij}|$$

#### Equations

In the remainder of this section, an exhaustive list of chemical reactions and equations for reaction rates used in the model is given. The data are segmented into reactions present in the cytosol, intermembrane space, mitochondrial matrix, extracellular matrix, and the blood vessels surrounding the cell.

### Cytosolic enzymes and reactions

#### ATP buffering

Creatine Kinase

(R1) ADP+CrP+H→ATP+Cr

(1) $${\text{J}}_{\text{CK}}=X_{CK}\cdot \left(K_{CK}\cdot {\left[\text{ADP}\right]}_{\text{c}}\cdot {\left[\text{CrP}\right]}_{\text{c}}\cdot {\left[\text{H}\right]}_{\text{c}}-{\left[\text{ATP}\right]}_{\text{c}}\cdot {\left[\text{Cr}\right]}_{\text{c}}\right)$$

**Table:** 

Parameter	Value	Unit	Source
$X_{CK}$	10,000	$\text{mM}/\left(\text{s}\cdot {\text{mM}}^{2}\right)$	(82)
$K_{CK}$	1,660,000	1/mM	(82)

Adenylate Kinase

(R2) 2ADP→AMP+ATP

(2) $${\text{J}}_{\text{AK}}=X_{AK}\cdot \left(K_{AK}\cdot {\left[\text{ADP}\right]}_{\text{c}}^{2}-{\left[\text{ATP}\right]}_{\text{c}}\cdot {\left[\text{AMP}\right]}_{\text{c}}\right)$$

**Table:** 

Parameter	Value	Unit	Source
$X_{AK}$	10,000	$\text{mM}/\left(\text{s}\cdot {\text{mM}}^{2}\right)$	(82)
$K_{AK}$	1	unitless	(27)

#### Energy consumption

ATP Consumption

(R3) ATP→ADP+Pi

(3) $${\text{J}}_{\text{ATPase}}=\frac{X_{ATPase}}{1+R\cdot \frac{{\left[\text{Pi}\right]}_{\text{c}}{\left[\text{ADP}\right]}_{\text{c}}}{{\left[\text{ATP}\right]}_{\text{c}}}}$$

**Table:** 

Parameter	Value	Unit	Source
$X_{ATPase}$	0.39	mM/s	(25)
*R*	65.8	1/mM	(25)

#### Glycolysis

Hexokinase

(R4) Glucose+ATP→G6P+ADP+H

(4) $${\text{J}}_{\text{HK}}=\frac{V_{f}\cdot \frac{{\left[\text{Glucose}\right]}_{\text{c}}\cdot {\left[\text{ATP}\right]}_{\text{c}}}{K_{m,f}\cdot \left({\left[\text{ATP}\right]}_{\text{c}}\;+\;K_{m,ATP}\right)}\;-V_{r}\cdot \frac{{\left[\text{G}6\text{P}\right]}_{\text{c}}}{K_{m,r}}}{1\;+\;\frac{{\left[\text{Glucose}\right]}_{\text{c}}}{K_{m,f}}\;+\;\frac{{\left[\text{G}6\text{P}\right]}_{\text{c}}}{K_{m,r}}}$$

**Table:** 

Parameter	Value	Unit	Source
$V_{f}$	0.550	mM/s	(24)
$K_{m,f}$	0.072	mM	(24)
$K_{m,ATP}$	0.236	mM	(24)
$V_{r}$	$1.06\times 10^{-4}$	mM/s	(24)
$K_{m,r}$	0.042	mM	(24)

Phosphoglucose Isomerase

(R5) G6P→F6P

(5) $${\text{J}}_{\text{PGI}}=\frac{V_{f}\cdot \frac{{\left[\text{G}6\text{P}\right]}_{\text{c}}}{K_{m,f}}\;-V_{r}\cdot \frac{{\left[\text{F}6\text{P}\right]}_{\text{c}}}{K_{m,r}}}{1\;+\;\frac{{\left[\text{G}6\text{P}\right]}_{\text{c}}}{K_{m,f}}\;+\;\frac{{\left[\text{F}6\text{P}\right]}_{\text{c}}}{K_{m,r}}}$$

**Table:** 

Parameter	Value	Unit	Source
$V_{f}$	10.067	mM/s	(24)
$K_{m,f}$	0.425	mM	(24)
$V_{r}$	9.6	mM/s	(24)
$K_{m,r}$	0.175	mM	(24)

Phosphofructokinase

(R6) F6P + ATP→F16P + ADP + H

(6) $${\text{J}}_{\text{PFK}}=V_{f}\cdot \frac{\frac{{\left[\text{F}6\text{P}\right]}_{\text{c}}}{K_{m,f}\;+\;{\left[\text{F}6\text{P}\right]}_{\text{c}}}}{1\;+\;\frac{K_{m,ATP}}{{\left[\text{ATP}\right]}_{\text{c}}}}$$

**Table:** 

Parameter	Value	Unit	Source
$V_{f}$	1.328	mM/s	(24)
$K_{m,f}$	0.224	mM	(24)
$K_{m,ATP}$	0.127	mM	(24)

Fructose-bisphosphate Aldolase

(R7) F16P + ATP→DHAP+GAP

(7) $${\text{J}}_{\text{FBPA}}=V_{f}\cdot \frac{{\left[\text{F}16\text{BP}\right]}_{\text{c}}}{{\left[\text{F}16\text{BP}\right]}_{\text{c}}\;+\;K_{m,f}}$$

**Table:** 

Parameter	Value	Unit	Source
$V_{f}$	0.992	mM/s	(24)
$K_{m,f}$	0.038	mM	(24)

Triosephosphate Isomerase

(R8) DHAP→GAP

(8) $${\text{J}}_{\text{TPI}}=V_{f}\cdot \frac{{\left[\text{DHAP}\right]}_{\text{c}}}{{\left[\text{DHAP}\right]}_{\text{c}}\;+\;K_{m,f}}$$

**Table:** 

Parameter	Value	Unit	Source
$V_{f}$	5.933	mM/s	(24)
$K_{m,f}$	1.53	mM	(24)

Glyceraldehyde 3-Phosphate Dehydrogenase

(R9) GAP+NAD+Pi→13BPG+NADH+H

(9) $${\text{J}}_{\text{GAPDH}}=V_{f}\cdot \frac{\frac{{\left[\text{GAP}\right]}_{\text{c}}}{K_{m,f}\;+\;{\left[\text{GAP}\right]}_{\text{c}}}}{1\;+\;\frac{K_{m,NAD}}{{\left[\text{NAD}\right]}_{\text{c}}}}$$

**Table:** 

Parameter	Value	Unit	Source
$V_{f}$	5.35	mM/s	(24)
$K_{m,f}$	0.042	mM	(24)
$K_{m,NAD}$	0.058	mM	(24)

Phosphoglycerate Kinase

(R10) 13BPG + ADP + H→3PG + ATP

(10) $${\text{J}}_{\text{PGK}}=\frac{V_{f}\cdot \frac{{\left[13\text{BPG}\right]}_{\text{c}}\cdot s_{1}\left({\left[13\text{BPG}\right]}_{\text{c}}\right)}{K_{m,f}\cdot \left(1+\frac{K_{m,ADP}}{{\left[\text{ADP}\right]}_{\text{c}}}\right)}\;-V_{r}\cdot \frac{{\left[3\text{PG}\right]}_{\text{c}}\cdot s_{1}\left({\left[3\text{PG}\right]}_{\text{c}}\right)}{K_{m,r}\cdot \left(1+\frac{K_{m,ATP}}{{\left[\text{ATP}\right]}_{\text{c}}}\right)}}{1\;+\;\frac{{\left[13\text{BPG}\right]}_{\text{c}}}{K_{m,f}}\;+\;\frac{{\left[3\text{PG}\right]}_{\text{c}}}{K_{m,r}}}$$

**Table:** 

Parameter	Value	Unit	Source
$V_{f}$	251	mM/s	(24)
$K_{m,f}$	0.021	mM	(24)
$K_{m,ADP}$	0.565	mM	(24)
$V_{r}$	15.98	mM/s	(24)
$K_{m,r}$	0.51	mM	(24)
$K_{m,ATP}$	0.008	mM	(24)

Phosphoglycerate Mutase

(R11) 3PG→2PG

(11) $${\text{J}}_{\text{PGM}}=\frac{V_{f}\cdot \frac{{\left[3\text{PG}\right]}_{\text{c}}\cdot s_{1}\left({\left[3\text{PG}\right]}_{\text{c}}\right)}{K_{m,f}}\;-V_{r}\cdot \frac{{\left[2\text{PG}\right]}_{\text{c}}\cdot s_{1}\left({\left[2\text{PG}\right]}_{\text{c}}\right)}{K_{m,r}}}{1\;+\;\frac{{\left[3\text{PG}\right]}_{\text{c}}}{K_{m,f}}\;+\;\frac{{\left[2\text{PG}\right]}_{\text{c}}}{K_{m,r}}}$$

**Table:** 

Parameter	Value	Unit	Source
$V_{f}$	11.233	mM/s	(24)
$K_{m,f}$	0.145	mM	(24)
$V_{r}$	48.0	mM/s	(24)
$K_{m,r}$	0.139	mM	(24)

Enolase

(R12) $$2\text{PG}\to \text{PEP}\;+\;{\text{H}}_{2}\text{O}$$

(12) $${\text{J}}_{\text{Enolase}}=\frac{V_{f}\cdot \frac{\left[2\text{PG}\right]\cdot s_{1}\left(\left[2\text{PG}\right]\right)}{K_{m,f}}\;-V_{r}\cdot \frac{\left[\text{PEP}\right]}{K_{m,r}}}{1\;+\;\frac{\left[2\text{PG}\right]}{K_{m,f}}\;+\;\frac{\left[\text{PEP}\right]}{K_{m,r}}}$$

**Table:** 

Parameter	Value	Unit	Source
$V_{f}$	1.85	mM/s	(24)
$K_{m,f}$	0.045	mM	(24)
$V_{r}$	2.00	mM/s	(24)
$K_{m,r}$	0.089	mM	(24)

Pyruvate Kinase

(R13) PEP+ADP→PYR+ATP

(13) $${\text{J}}_{\text{PK}}=\frac{V_{f}\cdot \frac{{\left[\text{PEP}\right]}_{\text{c}}\cdot s_{1}\left({\left[\text{PEP}\right]}_{\text{c}}\right)}{K_{m,f}\cdot \left(1\;+\;\frac{K_{m,ADP}}{{\left[\text{ADP}\right]}_{\text{c}}}\right)}\;-V_{r}\cdot \frac{{\left[\text{PYR}\right]}_{\text{c}}\cdot s_{1}\left({\left[\text{PYR}\right]}_{\text{c}}\right)}{K_{m,r}}}{1\;+\;\frac{{\left[\text{PEP}\right]}_{\text{c}}}{K_{m,f}}\;+\;\frac{{\left[\text{PYR}\right]}_{\text{c}}}{K_{m,r}}}$$

**Table:** 

Parameter	Value	Unit	Source
$V_{f}$	9.433	mM/s	(24)
$K_{m,f}$	0.11	mM	(24)
$K_{m,ADP}$	0.00268	mM	(22, 24)
$V_{r}$	0.00105	mM/s	(24)
$K_{m,r}$	10	mM	(24)

Glucose-6-Phosphate Dehydrogenase

(R14) G6P→ø

(14) $${\text{J}}_{\text{G}6\text{PDH}}=V_{f}\cdot \frac{1}{v_{ss,PGI}}\cdot {\text{J}}_{\text{PGI}}$$

**Table:** 

Parameter	Value	Unit	Source
$V_{f}$	0.095	mM/s	(24)
$v_{ss,PGI}$	0.125	mM/s	(24)

Glycerol-3-Phosphate Dehydrogenase

(R15) DHAP+NADH→Glycerol−3−P+NAD

(15) $${\text{J}}_{\text{G}3\text{PDH}}=V_{f}\frac{{\left[\text{DHAP}\right]}_{\text{c}}\cdot {\left[\text{NADH}\right]}_{\text{c}}}{K_{ia,NADH}\cdot K_{m,DHAP}\;+\;K_{m,DHAP}\cdot {\left[\text{NADH}\right]}_{\text{c}}\;+\;K_{m,NADH}\cdot {\left[\text{DHAP}\right]}_{\text{c}}\;+\;{\left[\text{DHAP}\right]}_{\text{c}}\cdot {\left[\text{NADH}\right]}_{\text{c}}}$$

**Table:** 

Parameter	Value	Unit	Source
$V_{f}$	0.095	mM/s	(22)
$K_{ia,NADH}$	0.095	mM	(83)
$K_{m,DHAP}$	0.095	mM	(83)
$K_{m,NADH}$	0.095	mM	(83)

Glucose-1,6-Bisphosphate Synthase

(R16) 13BPG+G1P→3PG+G16BP

(16) $${\text{J}}_{\text{G}16\text{BPS}}=\frac{{\text{V}}_{\text{f}}\cdot \frac{{\left[13\text{BPG}\right]}_{\text{c}}}{K_{m,f}\cdot \left(1\;+\;\frac{K_{m,G1P}}{{\left[\text{G}1\text{P}\right]}_{\text{c}}}\right)}\;-V_{r}\cdot \frac{{\left[3\text{PG}\right]}_{\text{c}}}{K_{m,r}\cdot \left(1\;+\;\frac{K_{m,G16BP}}{{\left[\text{G}16\text{BP}\right]}_{\text{c}}}\right)}}{1\;+\;\frac{{\left[13\text{BPG}\right]}_{\text{c}}}{K_{m,f}}\;+\;\frac{{\left[3\text{PG}\right]}_{\text{c}}}{K_{m,r}}}$$

**Table:** 

Parameter	Value	Unit	Source
$V_{f}$	10	mM/s	(22)
$K_{m,f}$	0.021	mM	(22)
$K_{m,G1P}$	0.008	mM	(22)
$V_{r}$	6	mM/s	(22)
$K_{m,r}$	0.51	mM	(22)
$K_{m,G16BP}$	0.565	mM	(22)

#### Glycogenesis and glycogenolysis

Phosphoglucomutase

(R17) G1P→G6P

(17) $${\text{J}}_{\text{PGluM}}=\frac{V_{f}\cdot \frac{{\left[\text{G}1\text{P}\right]}_{\text{c}}}{K_{m,f}}\;-V_{r}\cdot \frac{{\left[\text{G}6\text{P}\right]}_{\text{c}}}{K_{m,r}}}{1\;+\;\frac{{\left[\text{G}1\text{P}\right]}_{\text{c}}}{K_{m,f}}\;+\;\frac{{\left[\text{G}6\text{P}\right]}_{\text{c}}}{K_{m,r}}}$$

**Table:** 

Parameter	Value	Unit	Source
$V_{f}$	1.933	mM/s	(24)
$K_{m,f}$	0.045	mM	(24)
$V_{r}$	1.12	mM/s	(24)
$K_{m,r}$	0.67	mM	(24)

UDP-Glucose Pyrophosphorylase

(R18) G1P + UTP + H→UDPG+2Pi

(18) $${\text{J}}_{\text{UDPGP}}=X_{UDPGP}\cdot \frac{k_{f}\cdot {\left[\text{G}1\text{P}\right]}_{\text{c}}-{\left[\text{UDPG}\right]}_{\text{c}}\cdot k_{r}}{1+{\left(\frac{K_{m,AMP}}{{\left[\text{AMP}\right]}_{\text{c}}}\right)}^{1.5}}$$

**Table:** 

Parameter	Value	Unit	Source
$X_{UDPGP}$	10,000	unitless	(82)
$k_{f}$	4.36	1/s	(24)
$K_{m,AMP}$	0.016	mM	(84)
$k_{r}$	0.881	1/s	(24)

Glycogen Synthase (D/I Forms)

(R19) UDPG→UDP+Glycogen

(19) $${\text{J}}_{\text{GSD}}=V_{f,GSD}\cdot \frac{{\left[\text{UDPG}\right]}_{\text{c}}}{\left(K_{m,f,GSD}\;+\;{\left[\text{UDPG}\right]}_{\text{c}}\right)\cdot \left(1\;+\;{\left(\frac{K_{m,AMP}}{{\left[\text{AMP}\right]}_{\text{c}}}\right)}^{1.5}\right)}$$

(20) $${\text{J}}_{\text{GSI}}=V_{f,GSI}\cdot \frac{{\left[\text{UDPG}\right]}_{\text{c}}}{\left(K_{m,f,GSI}\;+\;{\left[\text{UDPG}\right]}_{\text{c}}\right)\cdot \left(1\;+\;{\left(\frac{K_{m,AMP}}{{\left[\text{AMP}\right]}_{\text{c}}}\right)}^{1.5}\right)}$$

**Table:** 

Parameter	Value	Unit	Source
$V_{f,GSD}$	0.147	mM/s	(24)
$K_{m,f,GSD}$	1.42	mM	(24)
$V_{f,GSI}$	0.147	mM/s	(24)
$K_{m,f,GSI}$	0.08	mM	(24)
$K_{m,AMP}$	0.016	mM	(84)

Glycogen Phosphorylase

(R20) Glycogen+Pi→G1P

(21) $${\text{J}}_{\text{GlyPhos}}=\frac{V_{f}\cdot \frac{{\left[\text{Glycogen}\right]}_{\text{c}}}{K_{m,f}}\;-V_{r}\cdot \frac{{\left[\text{G}1\text{P}\right]}_{\text{c}}}{K_{m,r}}}{\left(1\;+\;\frac{{\left[\text{Glycogen}\right]}_{\text{c}}}{K_{m,f}}\;+\;\frac{{\left[\text{G}1\text{P}\right]}_{\text{c}}}{K_{m,r}}\right)\cdot \left(1\;+\;{\left(\frac{K_{m,AMP}}{\left[\text{AMP}\right]}\right)}^{1.5}\right)}$$

**Table:** 

Parameter	Value	Unit	Source
$V_{f}$	0.782	mM/s	(24)
$K_{m,f}$	0.1	mM	(24)
$V_{r}$	55.83	mM/s	(24)
$K_{m,r}$	5	mM	(24)
$K_{m,AMP}$	0.016	mM	(84)

#### Lactate dynamics

Lactate Dehydrogenase

(R21) PYR+NADH+H→L+NAD

(22) $${\text{J}}_{\text{LDH}}=V_{f}\cdot \frac{\frac{\left[\text{PYR}\right]}{K_{m,f}+\left[\text{PYR}\right]}}{1\;+\;\frac{K_{m,NADH}}{\left[\text{NADH}\right]}}$$

**Table:** 

Parameter	Value	Unit	Source
$V_{f}$	23.93	mM/s	(24)
$K_{m,f}$	0.125	mM	(24)
$K_{m,NADH}$	0.001	mM	(24)

Lactate Hydrogen Cotransporter

(R22) $${\text{L}}_{e}+{\text{H}}_{e}\to \text{L}+\text{H}$$

(23) $${\text{J}}_{\text{LHX}}=V_{f}\cdot \frac{{\left[\text{L}\right]}_{\text{e}}}{{\left[\text{L}\right]}_{\text{e}}+K_{m,f}}\;-V_{r}\cdot \frac{{\left[\text{L}\right]}_{\text{c}}}{{\left[\text{L}\right]}_{\text{c}}+K_{m,r}}$$

**Table:** 

Parameter	Value	Unit	Source
$V_{f}$	0.048	mM/s	(27)
$K_{m,f}$	2.2	mM	(27)
$V_{r}$	0.182	mM/s	(27)
$K_{m,r}$	6.92	mM	(27)

Lactic Acid Association

(R23) L+H→LA

(24) $${\text{J}}_{\text{LA}}=X_{LA}\cdot \left(\frac{{\left[\text{L}\right]}_{\text{c}}\cdot {\left[\text{H}\right]}_{\text{c}}}{K_{m}}-{\left[\text{LA}\right]}_{\text{c}}\right)$$

**Table:** 

Parameter	Value	Unit	Source
$X_{LA}$	10,000	1/s	(27)
$K_{m}$	$1.259\times 10^{-7}$	mM	(27)

#### Other

Myoglobin-Oxygen Binding

(R24) $$\text{Mb}+{\text{O}}_{2}\to {\text{MbO}}_{2}$$

(25) $${\text{J}}_{\text{MB}}=k_{a}\cdot {\left[\text{Mb}\right]}_{\text{c}}\cdot {\left[{\text{O}}_{2}\right]}_{\text{c}}\;-\;k_{d}\cdot {\left[{\text{MbO}}_{2}\right]}_{\text{c}}$$

**Table:** 

Parameter	Value	Unit	Source
$k_{a}$	15,400	1/(mM⋅s)	(85)
$k_{b}$	60	1/s	(85)

Magnesium Binding

(R25) fADP+Mg→mADP

(26) $$\beta_{\text{ADP}}=\frac{1}{2}\cdot \left(K_{MgADP}+{\left[\text{Mg}\right]}_{\text{c},\text{tot}}+{\left[\text{mADP}\right]}_{\text{c}}-\sqrt{K_{MgADP}\;+\;{\left[\text{Mg}\right]}_{\text{c},\text{tot}}\;-\;4{\left[\text{mADP}\right]}_{\text{c}}{\left[\text{Mg}\right]}_{\text{c},\text{tot}}}\right)$$

**Table:** 

Parameter	Value	Unit	Source
$K_{MgADP}$	0.347	mM	(23, 86)
${\left[\text{Mg}\right]}_{\text{c},\text{tot}}$	5	mM	(23)

#### Mitochondrial intermembrane space

Magnesium Binding

(R26) fADP+Mg→mADP

(R27) fATP+Mg→mATP

(27) $${\text{J}}_{{\text{MgADP}}_{\text{i}}}=X_{J_{MgA}}\cdot \left({\left[\text{ADPf}\right]}_{\text{i}}\cdot {\left[\text{Mg}\right]}_{\text{i}}-K_{MgADP}\cdot {\left[\text{mADP}\right]}_{\text{i}}\right)$$

(28) $${\text{J}}_{{\text{MgATP}}_{\text{i}}}=X_{MgA}\cdot \left({\left[\text{fATP}\right]}_{\text{i}}\cdot {\left[\text{Mg}\right]}_{\text{i}}-K_{MgATP}\cdot {\left[\text{mATP}\right]}_{\text{i}}\right)$$

**Table:** 

Parameter	Value	Unit	Source
$X_{MgA}$	1000	1/(mM⋅s)	(23)
$K_{MgADP}$	0.347	mM	(23, 86)
$K_{MgATP}$	0.024	mM	(23, 86)

#### Phosphate transporter

Phosphate Hydrogen Cotransporter

(R28) $${\text{H}}_{2}{\text{PO}}_{4}\to {\text{HPO}}_{4}\;+\;\text{H}$$

(29) $${\text{J}}_{\text{Pi}1}=X_{Pi1}\cdot \frac{\left({\left[\text{H}\right]}_{\text{x}}\cdot {\left[{\text{H}}_{2}{\text{PO}}_{4}\right]}_{\text{i}}-{\left[\text{H}\right]}_{\text{i}}\cdot {\left[{\text{H}}_{2}{\text{PO}}_{4}\right]}_{\text{x}}\right)}{{\left[{\text{H}}_{2}{\text{PO}}_{4}\right]}_{\text{i}}\;+\;k_{PiHt}}$$

**Table:** 

Parameter	Value	Unit	Source
$X_{Pi1}$	3.394 × 10^5^	1/s	(23)
$k_{PiHt}$	0.4508	mM	(23)

Passive Phosphate Transport

(R29) $${\text{Pi}}_{x}\to {\text{Pi}}_{i}$$

(30) $${\text{J}}_{\text{Pi}2}=p_{Pi}\cdot \gamma \cdot \left({\left[\text{Pi}\right]}_{\text{x}}-{\left[\text{Pi}\right]}_{\text{i}}\right)$$

**Table:** 

Parameter	Value	Unit	Source
γ	5.99	1/μm	(23)
$p_{Pi}$	327	μm/s	(23)

Adenine Nucleotide Translocase

(R30) $${\text{ADPf}}_{i}+{\text{fATP}}_{m}\to {\text{fADP}}_{m}\;+\;{\text{fATP}}_{i}$$

(31) $${\text{J}}_{\text{ANT}}=X_{ANT}\cdot \left(\frac{{\left[\text{fADP}\right]}_{\text{i}}}{{\left[\text{fADP}\right]}_{\text{i}}+{\left[\text{fATP}\right]}_{\text{i}}\cdot e^{-\left(\frac{F}{RT}\right)\cdot 0.35\cdot \text{ΔΨ}}}-\frac{{\left[\text{fADP}\right]}_{\text{x}}}{{\left[\text{fADP}\right]}_{\text{x}}+{\left[\text{fATP}\right]}_{\text{x}}\cdot e^{\frac{F}{RT}\cdot 0.65\cdot \text{ΔΨ}}}\right)\cdot \frac{{\left[\text{fADP}\right]}_{\text{i}}}{{\left[\text{fADP}\right]}_{\text{i}}+K_{m,ADP}}$$

### Mitochondrial matrix

#### TCA cycle

Phenomenological equation for total TCA cycle

(R31) NAD+H→NADH

(32) $${\text{J}}_{\text{DH}}=X_{DH}\cdot \left(r\cdot {\left[\text{NAD}\right]}_{\text{x}}-{\left[\text{NADH}\right]}_{\text{x}}\right)\cdot \frac{1\;+\;\frac{{\left[\text{Pi}\right]}_{\text{x}}}{k_{Pi1}}}{1\;+\;\frac{{\left[\text{Pi}\right]}_{\text{x}}}{k_{Pi2}}}$$

**Table:** 

Parameter	Value	Unit	Source
$X_{DH}$	0.092	1/s	(23)
*r*	4.581	unitless	(23)
$k_{Pi,1}$	0.134	mM	(23)
$k_{Pi,2}$	0.677	mM	(23)

**Table:** 

Parameter	Value	Unit	Source
$X_{ANT}$	4.752	mM/s	a
$K_{m,ADP}$	3.5 × 10^−3^	mM	(23)

a The activity of ANT was optimized to 4.752 to fit the same experimental data as used by Beard (23).

#### Electron transport chain

Complex I

(R32) $$\text{H}+\text{NADH}\;+\;\text{Q}\to \text{NAD}\;+\;{\text{QH}}_{2}\;+\;4\text{ΔH}$$

(33) $${\text{J}}_{\text{C}1}=X_{C1}\cdot \left(e^{\frac{-\left(\text{Δ}{\text{G}}_{\text{C}1}+4\cdot \text{Δ}{\text{G}}_{\text{H}}\right)}{\text{RT}}}\cdot {\left[\text{NADH}\right]}_{\text{x}}-{\left[\text{NAD}\right]}_{\text{x}}\right)$$

(34) $$\text{Δ}{\text{G}}_{\text{C}1}=\text{Δ}{\text{G}}_{0,\text{C}1}\;-\;\text{RT}\cdot \text{ln}\left(\frac{{\left[\text{H}\right]}_{\text{x}}}{10^{-4}}\right)\;-\;\text{RT}\cdot \text{ln}\left(\frac{{\left[\text{Q}\right]}_{\text{x}}}{{\left[{\text{QH}}_{2}\right]}_{\text{x}}}\right)$$

**Table:** 

Parameter	Value	Unit	Source
$X_{C1}$	0.369	1/s	(23)
$\text{Δ}{\text{G}}_{0,\text{C}1}$	−69.37	kJ/mol	(23)

Complex III

(R33) $${\text{QH}}_{2}+2\text{cytC}\left(\text{ox}\right)\to \text{Q}\;+\;2\text{cytC}\left(\text{red}\right)\;+\;2\text{H}\;+\;4\text{ΔH}$$

(35) $${\text{J}}_{\text{C}3}=X_{C3}\cdot \left(e^{\frac{-0.5\cdot \left(\text{Δ}{\text{G}}_{\text{C}3}+4\cdot \text{Δ}{\text{G}}_{\text{H}}-F\cdot \text{ΔΨ}\cdot 2\right)}{\text{RT}}}\cdot {\left[\text{Cox}\right]}_{\text{i}}-{\left[\text{Cred}\right]}_{\text{i}}\right)\cdot \frac{1\;+\;\frac{{\left[\text{Pi}\right]}_{\text{x}}}{k_{Pi,1}}}{1\;+\;\frac{{\left[\text{Pi}\right]}_{\text{x}}}{k_{Pi,2}}}$$

(36) $$\text{Δ}{\text{G}}_{\text{C}3}=\text{Δ}{\text{G}}_{0,\text{C}3}\;+\;\text{RT}\cdot \text{ln}\left(\frac{{\left[\text{H}\right]}_{\text{x}}}{10^{-4}}\right)\;-\;\text{RT}\cdot \text{ln}\left(\frac{{\left[{\text{QH}}_{2}\right]}_{\text{x}}}{{\left[\text{Q}\right]}_{\text{x}}}\right)$$

**Table:** 

Parameter	Value	Unit	Source
$X_{C3}$	0.092	1/s	(23)
$k_{Pi,1}$	0.192	mM	(23)
$k_{Pi,2}$	25.31	mM	(23)
$\text{Δ}{\text{G}}_{0,\text{C}1}$	−32.53	kJ/mol	(23)

Complex IV

(R34) $$2\text{H}+2\text{cytC}\left(\text{red}\right)+1/2{\text{O}}_{2}\to 2\text{cytC}\left(\text{ox}\right)+{\text{H}}_{2}\text{O}+2\text{ΔH}$$

(37) $${\text{J}}_{\text{C}4}={\text{X}}_{\text{C}4}\cdot \frac{1}{1\;+\;k_{O_{2}}/\left[\text{O}2\right]}\cdot \frac{{\left[\text{Cred}\right]}_{\text{i}}}{{\left[\text{Cred}\right]}_{\text{i}}\;+\;{\left[\text{Cox}\right]}_{\text{i}}}\cdot \left(e^{\frac{-0.5\cdot \left(\text{Δ}{\text{G}}_{\text{C}4}+2\cdot \text{Δ}{\text{G}}_{\text{H}}\right)}{\text{RT}}}\cdot {\left[\text{Cred}\right]}_{\text{i}}\cdot {\left[\text{O}2\right]}_{\text{c}}^{0.25}-{\left[\text{Cox}\right]}_{\text{i}}\cdot e^{\frac{F}{RT}\cdot \text{ΔΨ}}\right)$$

(38) $$\text{Δ}{\text{G}}_{\text{C}4}=\text{Δ}{\text{G}}_{0,\text{C}4}-2\text{RT}\cdot \text{ln}\left(\frac{{\left[\text{H}\right]}_{\text{x}}}{10^{-4}}\right)-\frac{\text{RT}}{2}\cdot \text{ln}\left(\frac{\left[{\text{O}}_{2}\right]\cdot 1e-3}{1}\right)$$

F1F0-ATP Synthase

(R35) H+ADP+Pi+3ΔH→ATP

(39) $${\text{J}}_{\text{F}1}=X_{F1}\cdot \left(e^{\frac{-\left(\text{Δ}G_{0,F1}-3\cdot \text{Δ}{\text{G}}_{\text{H}}\right)}{\text{RT}}}\cdot \frac{K_{MgADP}}{K_{MgATP}}\cdot {\left[\text{mADP}\right]}_{\text{x}}\cdot {\left[\text{Pi}\right]}_{\text{x}}-\left(1000\left[\text{mM}\right]\right)\cdot {\left[\text{mATP}\right]}_{\text{x}}\right)$$

(40) $$\alpha_{\text{F}1}=\frac{{\left[\text{fATP}\right]}_{\text{x}}}{{\left[\text{ATP}\right]}_{\text{x}}}\cdot \frac{{\text{V}}_{\text{cyto}}}{{\text{V}}_{\text{mito}}}$$

**Table:** 

Parameter	Value	Unit	Source
$X_{F1}$	2.267 × 10^−5^	1/s	(23)
$K_{MgADP}$	3.47 × 10^−4^	mM	(86)
$K_{MgATP}$	2.45 × 10^−5^	mM	(86)
$\text{Δ}{\text{G}}_{0,\text{F}1}$	36.03	kJ/mol	(23)

**Table:** 

Parameter	Value	Unit	Source
$X_{C4}$	2.267 × 10^−5^	1/s	(23)
$k_{O_{2}}$	3	mM	a
$\text{Δ}{\text{G}}_{0,\text{C}4}$	−122.94	kJ/mol	(23)

a $k_{O_{2}}$ was optimized to 3 to fit the same experimental data as used by Beard (23).

Proton Leak

(R36) $${\text{H}}_{i}\to {\text{H}}_{m}$$

(41) $${\text{J}}_{\text{Hle}}=X_{Hle}\cdot \text{ΔΨ}\cdot \frac{{\left[\text{H}\right]}_{\text{i}}\cdot e^{\frac{F}{RT}\cdot \text{ΔΨ}}\;-\;{\left[\text{H}\right]}_{\text{x}}}{e^{\frac{F}{RT}\cdot \text{ΔΨ}}\;-\;1}$$

**Table:** 

Parameter	Value	Unit	Source
$X_{Hle}$	250	1/(s⋅mV)	(23)

#### Other

Proton Motive Energy

(42) $$\text{Δ}{\text{G}}_{\text{H}}=\text{F}\cdot \text{ΔΨ}\;+\;\text{RT}\cdot \text{ln}\left(\frac{{\left[\text{H}\right]}_{\text{i}}}{{\left[\text{H}\right]}_{\text{x}}}\right)$$

**Table:** 

Parameter	Value	Unit	Name
F	0.096484	(Ms⋅A)/mol	Faraday’s Constant
R	8.314	J/(mol⋅K)	Gas Constant
T	310	K	Temperature

Magnesium Binding

(R37) fADP+Mg→mADP

(43) $${\text{J}}_{{\text{MgADP}}_{\text{x}}}=X_{J_{MgA}}\cdot \left({\left[\text{fADP}\right]}_{\text{x}}\cdot {\left[\text{Mg}\right]}_{\text{x}}-K_{MgADP}\cdot {\left[\text{mADP}\right]}_{\text{x}}\right)$$

(44) $${\text{J}}_{{\text{MgATP}}_{\text{x}}}=X_{MgA}\cdot \left({\left[\text{fATP}\right]}_{\text{x}}\cdot {\left[\text{Mg}\right]}_{\text{x}}-K_{MgATP}\cdot {\left[\text{mATP}\right]}_{\text{x}}\right)$$

**Table:** 

Parameter	Value	Unit	Source
$X_{MgA}$	1000	1/(mM⋅s)	(23)
$K_{MgADP}$	0.347	mM	(23, 86)
$K_{MgATP}$	0.024	mM	(23, 86)

Potassium Hydrogen Transport

(R38) $${\text{K}}_{i}+{\text{H}}_{x}\to {\text{K}}_{x}\;+\;{\text{H}}_{i}$$

(45) $${\text{J}}_{\text{KH}}={\text{X}}_{\text{KH}}\cdot \left({\left[\text{K}\right]}_{\text{i}}\cdot {\left[\text{H}\right]}_{\text{x}}-{\left[\text{K}\right]}_{\text{x}}\cdot {\left[\text{H}\right]}_{\text{i}}\right)$$

**Table:** 

Parameter	Value	Unit	Source
$X_{KH}$	29,802	1/(mM⋅s)	(23)

### Remaining compartments

#### Oxygen transport

Oxygen Transport Vessel—Extracellular Matrix

(R39) $${\text{O}}_{2v}\to {\text{O}}_{2e}$$

(46) $${\text{J}}_{{\text{O}}_{2},\text{VE}}=p_{A}\cdot \gamma \cdot \left({\left[\text{O}2\right]}_{\text{v}}-{\left[\text{O}2\right]}_{\text{e}}\right)$$

**Table:** 

Parameter	Value	Unit	Source
$p_{A}$	50	l/s	(82)
γ	0.284	1/l	(22, 82)

Oxygen Transport Extracellular Matrix—Cell

(R40) $${\text{O}}_{2e}\to {\text{O}}_{2c}$$

(47) $${\text{J}}_{{\text{O}}_{2},\text{EC}}=p_{A}\cdot \gamma \cdot \left({\left[\text{O}2\right]}_{\text{e}}-{\left[\text{O}2\right]}_{\text{c}}\right)$$

**Table:** 

Parameter	Value	Unit	Source
$p_{A}$	10	l/s	(82)
γ	0.241	1/l	(22, 82)

## Data availability

The biological model consists of a collection of ordinary differential equations with rate constants that have been derived from the literature. They are described using the Systems Biology Markup Language and freely available on http://www.ebi/ac/uk/biomodels/.

## Conflict of interest

The authors declare that they have no conflicts of interest with the contents of this article.
